# Development of a covalent cereblon-based PROTAC employing a fluorosulfate warhead†

**DOI:** 10.1039/d3cb00103b

**Published:** 2023-08-31

**Authors:** Radosław P. Nowak, Leah Ragosta, Fidel Huerta, Hu Liu, Scott B. Ficarro, Justin T. Cruite, Rebecca J. Metivier, Katherine A. Donovan, Jarrod A. Marto, Eric S. Fischer, Breanna L. Zerfas, Lyn H. Jones

**Affiliations:** a Center for Protein Degradation, Dana-Farber Cancer Institute Boston MA USA lyn_jones@dfci.harvard.edu nowak@crystal.harvard.edu; b Department of Biological Chemistry and Molecular Pharmacology, Harvard Medical School Boston MA USA; c Department of Cancer Biology, Dana-Farber Cancer Institute Boston MA USA; d Department of Cancer Biology, Department of Oncologic Pathology, and Blais Proteomics Center, and Center for Emergent Drug Targets, Dana-Farber Cancer Institute Boston MA USA; e Department of Pathology, Brigham and Women's Hospital, Harvard Medical School Boston MA 02115 USA

## Abstract

Many cereblon (CRBN) ligands have been used to develop proteolysis targeting chimeras (PROTACs), but all are reversible binders of the E3 ubiquitin ligase. We recently described the use of sulfonyl exchange chemistry to design binders that covalently engage histidine 353 in CRBN for the first time. Here we show that covalent CRBN ligands can be used to develop efficient PROTAC degraders. We demonstrate that the fluorosulfate PROTAC FS-ARV-825 covalently labels CRBN *in vitro*, and in cells the BRD4 degrader is insensitive to wash-out and competition by potent reversible CRBN ligands, reflecting enhanced pharmacodynamics. We anticipate that covalent CRBN-based PROTACs will enhance degradation efficiencies, thus expanding the scope of addressable targets using the heterobifunctional degrader modality.

## Abstract 2

The first covalent cereblon-based PROTAC was developed using a fluorosulfate warhead to site-specifically engage His353 in the sensor loop of the E3 ligase, yielding a degrader with prolonged pharmacodynamics.

## Graphical abstract

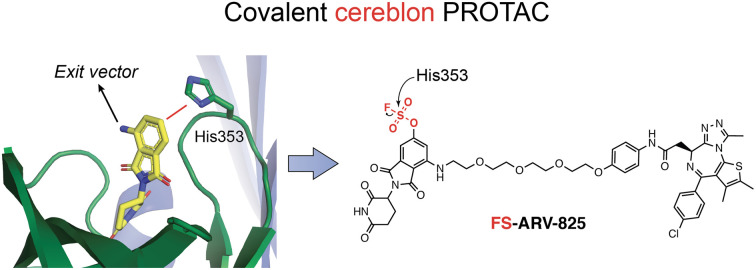

## Introduction

Targeted protein degradation is a promising therapeutic modality which relies on catalytic protein turnover accelerated by small molecule-mediated recruitment of the target proteins to the E3 ubiquitin ligase. Clinical utility of targeted protein degradation has been validated by approval of molecular glue degraders such as immunomodulatory imide drugs (IMiDs) thalidomide, lenalidomide and pomalidomide. On a molecular level IMiDs bind the substrate receptor cereblon (encoded by the gene CRBN) of the CRL4^CRBN^ E3 ubiquitin ligase and redirect it to degrade IKZF1/3 and CK1α, key dependencies in blood cancers.^1,2^ An alternative strategy to degrade proteins is to generate a bifunctional molecule, where an E3 ligase ligand is tethered to a target binder with a chemical linker creating a proteolysis targeting chimera (PROTAC).^3^ Both PROTACs and molecular glue degraders alter the interactome of the E3 ligase to induce recruitment and ubiquitination of specific target proteins (neosubstrates). Not only is the ubiquitination reaction catalytic, but also a single degrader molecule may be recycled to engage another target protein molecule and result in its efficient polyubiquitination and proteasomal degradation.^4^ Using mathematical simulations, it was suggested recently that covalent engagement of the E3 ligase will enhance catalytic efficiency by improving the kinetics of ternary complex formation and degradation.^5^ Therefore, covalent PROTACs may achieve the desired levels of target degradation with only fractional site occupancy of the E3.^6,7^ Such molecules are then less likely to perturb the endogenous functions of the E3 ligase.

Chemoproteomics studies have recently identified several small molecule covalent binders that engage cysteine residues on E3 ligases such as DCAF11, DCAF16, RNF4 and RNF114, and demonstrated that these can be developed into active PROTAC molecules.^8–13^ These proof-of-principle molecules rely on covalent ligands which are often broadly reactive, increasing the possibility of off-target binding, and increase complexity of the pharmacology of the PROTACs that incorporate them. Cereblon is one of the most widely used E3 ubiquitin ligases in the targeted protein degradation space, and most degraders in clinical trials are overwhelmingly CRBN modulators. However, there are currently no reports of PROTACs that take advantage of covalent CRBN ligands since there are no targetable cysteines within the IMiD binding site.^14^

Pioneering research by Baker and Colman demonstrated the potential of incorporating sulfonyl fluoride electrophiles into small molecule ligands and metabolites to create irreversible inhibitors and chemical biology probes that were shown to engage a variety of amino acid residues beyond cysteine in protein binding sites.^15,16^ Subsequently, several studies have successfully deployed sulfonyl fluorides, fluorosulfates and other related sulfur(vi) fluoride exchange (SuFEx) warheads to site-specifically label tyrosine, lysine, serine, and threonine residues across a wide variety of proteins.^17–22^ Histidine targeting is underexplored in covalent drug discovery, though SuFEx has been shown previously to be suitable for crosslinking proteins.^23–27^ We recently described the design of fluorosulfate (FS) and sulfonyl fluoride (SF) analogs of the IMiD EM12 using SuFEx chemistry, to covalently target a histidine residue (His353) in cereblon for the first time.^28^ Interestingly, while both molecules engage cereblon in cells, only EM12-FS retains molecular glue activity, and degrades the novel neosubstrate NTAQ1, while EM12-SO_2_F appears to lack degradation effects, yet displays subnanomolar engagement of cereblon in cells. The less reactive EM12-FS is capable of engaging cereblon in cells with an IC_50_ of 256 nM and maintains long plasma stability >196 min, with human hepatocyte *T*_1/2_ > 217 min and high human liver microsome stability *T*_1/2_ > 145 min, comparable to clinical IMiD molecules. These favourable characteristics suggest that fluorosulfate warheads have adequate stability for covalent drug design.^28^ Building on these proof-of-concept studies we asked whether a covalent cereblon binder can be used as an E3 ligase ligand for development of a PROTAC molecule.

We reasoned that the resulting covalent CRBN PROTAC would enhance degradation efficiency and expand the scope of addressable targets using targeted protein degradation. We also anticipate that the fractional occupancy of covalent E3 ligase modulators could lead to greater mutational resilience and be less dependent on expression levels of the ligase, since E3 downregulation is a known escape mechanism of anticancer CRBN modulators in the clinic.^29^ Here we established a proof-of-principle covalent PROTAC molecule targeting BRD4 by modifying the phthalimide-based ARV-825 to include a fluorosulfate covalent motif.^30^ We show that FS-ARV-825 covalently labels cereblon, degrades BRD4, and maintains its degradation activity even after washout of the probe indicating a covalent mode of action in cells.^31^

## Results and discussion

**Fig. 1:**
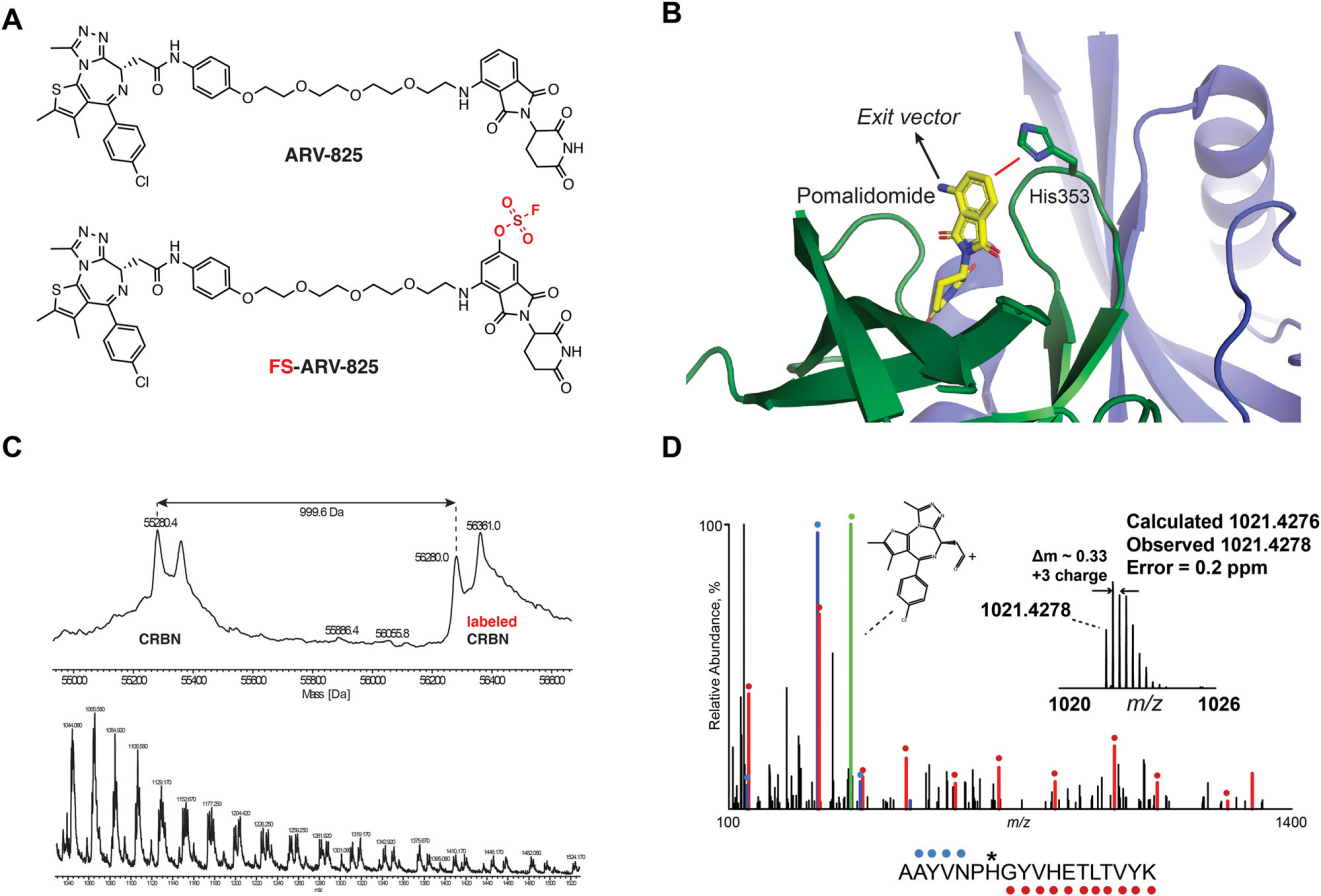
(A) Chemical structures of ARV-824 and its covalent fluorosulfate version FS-ARV-825. (B) Crystal structure of pomalidomide bound to CRBN (PDB: 4CI3) indicating the exit vector position. Proximity of His353 and pomalidomide is indicated by a red line. (C) Intact mass of cereblon indicating covalent labelling with FS-ARV-825. CRBN-DDB1ΔB and FS-ARV-825, both at 10 μM were incubated for 24 h at room temperature followed by intact protein MS analysis. Deconvoluted mass spectra as well as *m*/*z* ratios as depicted. (D) Mapping of the labelled site by MS/MS analysis. FS-ARV-825 modified CRBN tryptic peptide sequence (*m*/*z* = 1021.4276) with detected b- and y-type fragment ions indicated with blue and red glyphs, respectively. Internal fragmentation of FS-ARV-825 at the amide bond adjacent to the JQ1 moiety resulted in a structure-specific diagnostic ion at *m*/*z* = 383.0728 (green), which further confirmed covalent modification of His353 by FS-ARV-825.

We previously designed a series of covalent CRBN modulators to engage His353 in the sensor loop of the protein. Derivatives included a highly reactive sulfonyl fluoride (EM12-SO_2_F), which displayed low plasma stability, and a fluorosulfate (EM12-FS) with excellent human plasma microsome and hepatocyte stability. We reasoned that incorporation of the fluorosulfate warhead into an existing PROTAC such as the BRD4 targeting ARV-825 would deliver a covalent PROTAC.^30^ We prepared FS-ARV-825 which contains a fluorosulfate group in the 6-position of the phthalimide ring that was designed to engage His353 (Fig. 1A and B). The CRBN-DDB1ΔB protein complex was incubated with FS-ARV-825 and intact mass spectrometry (MS) confirmed covalent labelling (mass shift of 999.6 Da). Cereblon was ∼50% labelled under these conditions (Fig. 1C), and we did not observe any modification of DDB1ΔB, suggesting FS-ARV-825 selectively engages cereblon (Fig. S1A, ESI†). To verify the labelling site on CRBN we performed MS/MS analysis and mapped the labelled peptide to AAYVNPH*GYVHETLTVYK ([M + 3H]^3+^ = 1021.4278), confirming that His353 of CRBN is indeed the site of covalent modification (Fig. 1D). Interestingly, when analysing the MS/MS spectrum we noticed an intense ion at *m*/*z* = 383.0728, which corresponds to the JQ1 side of FS-ARV-825 fragmented at the amide bond (theoretical MW 1021.4276 *m*/*z*) (Fig. 1D, green). These data extend our previous work in identifying structure-specific fragment ions of covalent inhibitors and probes we leveraged these as diagnostic ions to improve confidence in peptide sequence assignment and site of modification.^32,33^

Having established covalent labelling using recombinant proteins, we measured the cellular engagement of cereblon with FS-ARV825 in HEK293T cells. We used a previously developed NanoBRET engagement assay which monitors competitive displacement of the BODIPY-FL-lenalidomide probe.^34^ We observed that the BODIPY-FL fluorophore forms an efficient BRET pair with a NanoLuc luciferase resulting in a BRET signal in the 450/520 nm range.^34^ The assay was first run after 2 h incubation with the compounds (Fig. S2A, ESI†). We observed that ARV-825 engages cereblon with an IC_50_ of 57 ± 8 nM (mean ± standard error), which is a 5-fold improvement over lenalidomide (IC_50_ 395 ± 39 nM). FS-ARV-825 displayed a reduced engagement following 2 h incubation in cells, with an IC_50_ of 850 ± 140 nM, 15-fold lower than its non-covalent congener ARV-825, but only two-fold weaker than lenalidomide. To further characterize the time component of cereblon engagement we performed the assay using live cell substrate, which enabled real time kinetic measurement of cereblon engagement in cells. The displacement of the BODIPY-FL-lenalidomide was observed to stabilize within the measurement timeframe of ∼2–3 min for ARV-825, while FS-ARV-825 continued to equilibrate, with the IC_50_ reaching steady state after 20–30 min after addition of the molecule, consistent with its covalent mode of action and low intrinsic reactivity (Fig. S2B, ESI†).

**Fig. 2:**
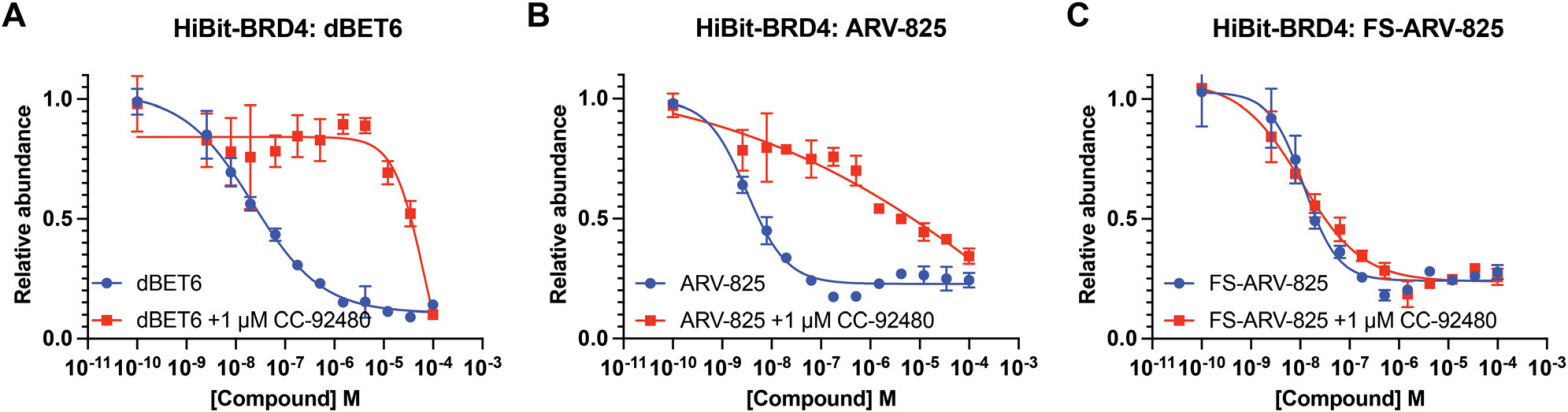
(A)–(C) HiBiT-BRD4 degradation assay. HEK293T cells stably expressing HiBiT-SpyTag-BRD4 were pre-treated with 1 μM of CC-92480 for 2 h and followed by PROTAC treatment for 24 h. Data are shown as mean ± s.d. of two replicates (*N* = 2).

**Fig. 3:**
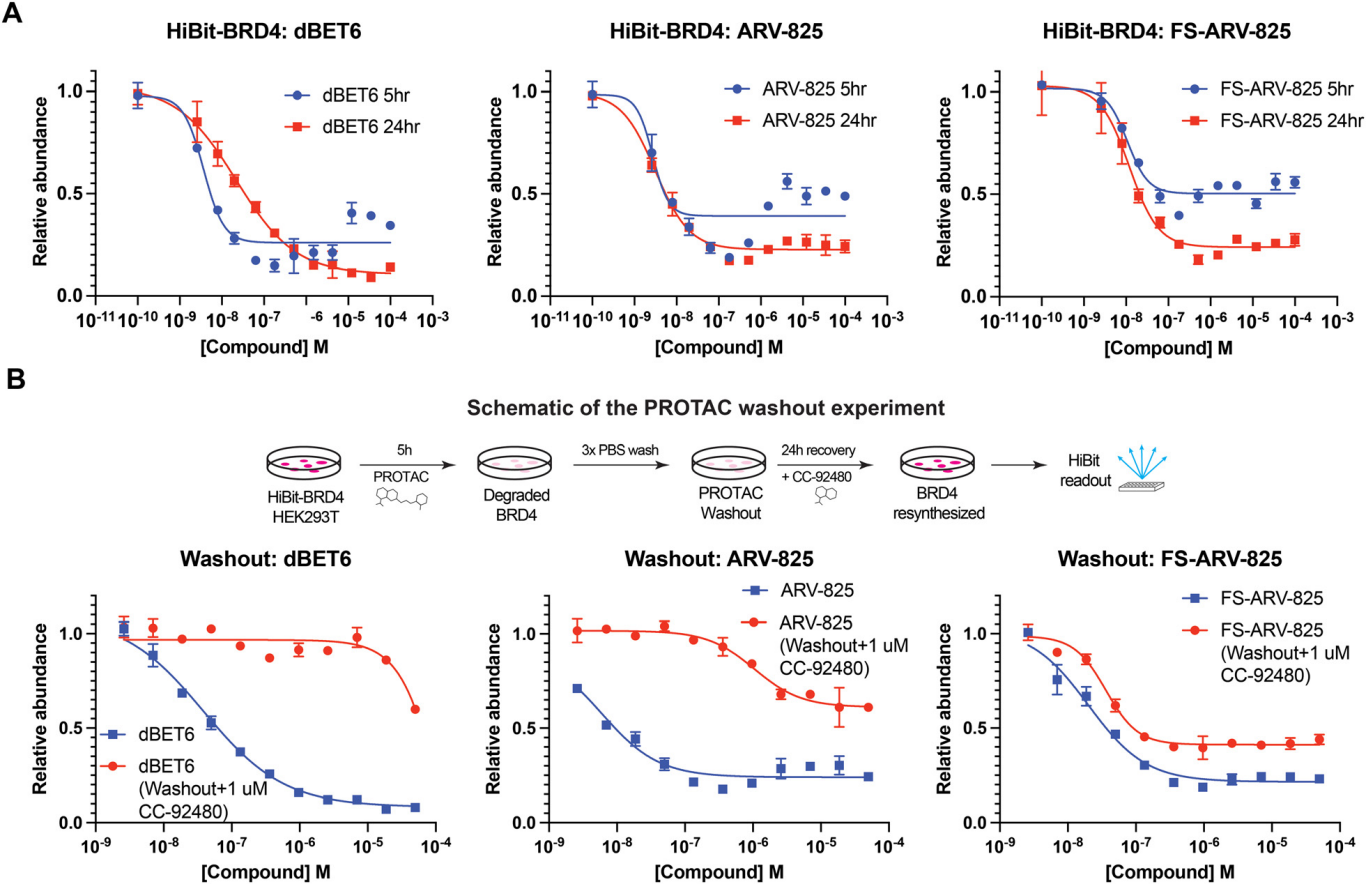
(A) HiBiT-BRD4 degradation assay. HEK293T cells stably expressing HiBiT-SpyTag-BRD4 were treated with PROTAC molecules for 5 or 24 h following a HiBiT lytic assay. Data are shown as mean ± s.d. of two replicates (*N* = 2). (B) Washout and recovery experiment. HEK293T cells stably expressing HiBiT-SpyTag-BRD4 were treated with a dose response of PROTAC degraders for 5 h followed by PBS wash and 24 h recovery in DMEM media with 10% FBS supplemented with 1 μM of CC-92480. CC-92480 is added in to block CRBN activity. The control plate was run as described above, but no washout was applied making the total incubation time 29 h (5 h + 24 h). Data are shown as mean ± s.d. of two replicates (*N* = 2).

We next tested the degradation activity of the FS-ARV-825 on endogenous BRD4 in HiBiT-SpyTag002-BRD4 HEK293T cells engineered with CRISPR-Cas9.^35^ We treated HiBiT-SpyTag002-BRD4 HEK293T cells with FS-ARV-825 and observed dose dependent degradation at the 5 h time point and degradation continued to increase at 24 h, while the non-covalent analog ARV-825 showed a similar degradation profile at 5 h and 24 h (Fig. 3A). The slower degradation kinetics of FS-ARV-825 is consistent with the intrinsic reactivity of the fluorosulfate warhead. To further confirm the covalent mode of action of FS-ARV-825 we pre-incubated cells for 2 h with 1 μM CC-92480, a recently described nanomolar affinity cereblon binder, followed by PROTAC treatment for 24 h (Fig. 2).^31^

Pre-treatment with CC-92480 inhibited CRBN and significantly reduced degradation by reversible molecules dBET6 and ARV-825 but had no impact on degradation by covalent FS-ARV-825, consistent with its covalent mode of action (Fig. 2). We next asked if a covalent cereblon PROTAC would be able to continue to degrade its target even after removal of the chemical probe. To address this question, we performed a washout experiment (Fig. 3B). HiBiT-SpyTag-BRD4 HEK293T cells were treated with a range of doses of FS-ARV-825, its non-covalent analog ARV-825 and dBET6 for 5 h, washed the cells with PBS to remove the compounds and incubated the cells with cell media supplemented with 1 μM of CC-92480 and allowed for resynthesis of BRD4 for 24 h. CC-92480 was used in this experiment to inactivate any residual PROTAC molecules that may not have been removed by the washout (Fig. 3B). The wash step was omitted in the control plate making the total incubation with the drug of 29 h (5 h + 24 h). We noticed that a 24 h recovery after washout of dBET6 or ARV-825 was sufficient to increase BRD4 protein levels to DMSO levels but strikingly, had nearly no effect on the FS-ARV-825 (Fig. 3B). The covalent FS-ARV-825 was still able to effectively degrade BRD4 with a similar inflection point albeit reduced *D*_max_.

**Fig. 4:**
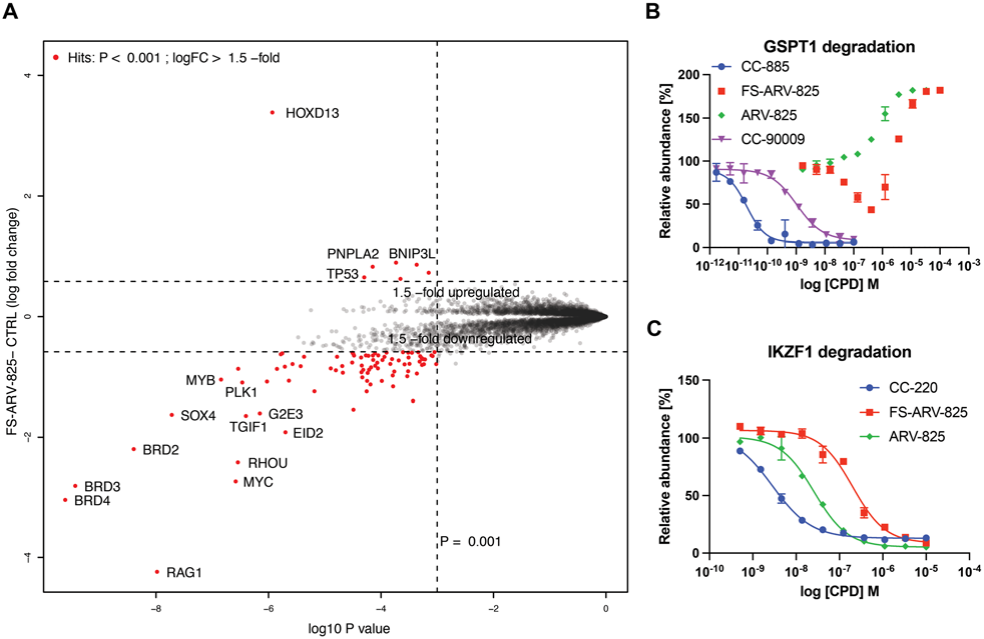
(A) Degradation proteomics. 5 h treatment of MOLT4 cells with 1 μM of FS-ARV-825 was followed up by proteomics assessment of protein abundance. (B) GSPT1 degradation. HEK293T cells expressing GFP-GSPT1 fusion with mCherry reporter were treated for 5 h following by assessment of GFP/mCherry ratio using high content imager Operetta. Data are shown as mean ± s.d. of two replicates (*N* = 2). (C) IKZF1 degradation. MOLT4 HiBiT-IKZF1 cells were treated for 24 h following by assessment protein levels using HiBiT lytic luminescence assay. Data are shown as mean ± s.d. of two replicates (*N* = 2).

To gain insight into the global selectivity profile of FS-ARV-825 we performed a proteomics experiment in MOLT4 cells treated with 1 μM of the compound for 5 h (Fig. 4A). The fluorosulfate molecular glue EM12-FS was shown previously to degrade the N-terminal glutamine hydrolase NTAQ1, but this target was not degraded in the proteomics profile of FS-ARV-825. FS-ARV-825 induced potent degradation of multiple proteins including BRD2, BRD3 and BRD4 indicating active PROTAC function on a broad family of BET bromodomains. Consistent with previous reports for ARV-825 and dBET6 we also observed downregulation of c-Myc and SOX4.^36,37^ In fact, the proteomics experiment identified many downregulated proteins compared to the DMSO sample, a profile consistent with the known BET bromodomain functions as master regulators of global transcription elongation.^37^ To validate the impact on cell viability we performed a CellTiter-Glo (CTG) assay in MOLT4 cells and observed a loss of viability that was similar for FS-ARV-825 and ARV-825 (Fig. S3, ESI†). CRBN based PROTACs frequently possess molecular glue activities, recruiting neosubstrates such as transcription factors IKZF1/3 or the translation termination factor GSPT1.^38,39^ IKZF1 together with BET bromodomains have recently been identified as interactors of ARV-825 PROTAC in AirID biotin enrichment proteomics experiment.^40^ Interestingly, we did not observe significant degradation of IKZF1 or GSPT1 by FS-ARV-825 in the proteomics experiment in MOLT4 cells. To quantitatively assess the potential for IMiD neosubstrate degradation we then profiled both molecules for degradation of GSPT1 and IKZF1 (Fig. 4B and C).^41^ GSPT1 degradation was measured after 5 h incubation of the degrader molecules in Flp293T cells stably expressing GFP-GSPT1_domain 3 (389-499)_/mCherry by quantification of GFP/mCherry ratio using an imaging-based method, an assay that we have found to be very sensitive.^42^ FS-ARV-825 was capable of degrading GSPT1 with a DC_50_ ∼ 500 nM, however autofluorescence at higher concentrations masked the full dose response. We observed a similar autofluorescence effect for ARV-825, which lacked any indication of GSPT1 degradation. The extended π-delocalization and push–pull electronics of the amino phthalimide moiety likely drives the autofluorescence.^38^ We also incubated MOLT4 cells harbouring N-terminal HiBiT knock in on IKZF1 with degrader molecules for 24 h. ARV-825 degraded IKZF1 with a DC_50, 24h_ of 26 ± 2 nM, while FS-ARV-825 was nearly 8-fold weaker with the DC_50, 24h_ of 200 ± 28 nM, both molecules achieving ∼95% *D*_max, 24h_. The IKZF1/3 degrader CC-220 currently in clinical trials provided a DC_50_ of 2.8 ± 2.8 nM.^43^ The significantly weaker molecular glue activity of FS-ARV-825 compared to ARV-825 is likely due to substitution of the 6-position of the phthalimide ring that will clash with the structural G-loop degron of neosubstrates.^39^

## Conclusions

We describe the development of a bromodomain-targeted PROTAC molecule utilizing covalent engagement of cereblon through sulfur(vi) fluoride exchange chemistry. We appreciate that the BRD4 pilot study here is the first step to characterize the breadth of utility of this covalent degradation modality more completely. The design of FS-ARV-825 was inspired by our recent discovery of small molecule covalent cereblon modulators employing fluorosulfate and sulfonyl fluoride covalent warheads.^28^ The fluorosulfate FS-ARV-825 covalently modified cereblon at His353 *in vitro* with 50% labelling efficiency observed after 24 h incubation, consistent with the low intrinsic reactivity of the fluorosulfate warhead. Further optimization of equilibrium binding interactions would likely enhance the rate of covalent labelling that could accelerate the degradation kinetics. We previously demonstrated that covalent inhibitors and probes undergo predictable internal fragmentation during MS/MS.^32,33^ The resulting structure-specific diagnostic ions can be used to improve confidence in peptide sequence assignment as well as to build targeted MS/MS acquisition schemes such as precursor ion scanning.^32^ Our detailed analytical characterization herein extends these concepts to heterobifunctional compounds comprising a single covalent warhead and may provide an efficient method to identify off-target labelling of covalent PROTACs in complex proteomes. We showed that covalent modification of cereblon resulted in sustained degradation of BRD4 even after probe washout, indicating that the covalent mode of action may present additional advantages for the targeted protein degradation modality such as enhanced catalytic efficiency. Our data also show that the covalent PROTAC FS-ARV-825 possesses lower CBRN occupancy in cells yet similar degradation potency to the reversible binding PROTAC ARV-825. Indeed, fractional occupancy of new E3 ligases using covalent degraders, and thus hijacking of a small percentage of the protein, may perturb the endogenous function of the E3 to a lesser extent, and enhance resilience to binding site mutations and potential downregulation of the E3 in cancer. While we observed FS-ARV-825 to be an active, albeit rather weak, IKZF1 and GSPT1 degrader, it is likely that additional modifications of the cereblon ligand would enable further tuning of these properties, to either impart additional functionality and efficacy, or to avoid neosubstrate off-targets and hence improve safety profiles.^39^ We hope that our reported ability to combine covalent engagement and catalytic mode of action in a widely used E3 ligase can provide an additional resource for the targeted protein degradation field. Although several PROTACs were developed previously that engage an E3 cysteine, the amino acid is rarely available for targeting within protein binding sites. Our work highlights the potential to expand the chemical toolbox of useful synthetic modifications that enable re-engineering and recruitment of a broader set of E3 ligases through targeting of residues beyond cysteine.

## Author contributions

RPN, BLZ, LR, FH, ESF, and JTC performed and/or designed the biochemical assays; KAD, SBF, JAM and RJM performed the mass spectrometry studies; LHJ, HL designed the compounds; LHJ conceived and directed the project; RPN and LHJ wrote the manuscript with contributions from all authors.

## Conflicts of interest

LHJ serves on the SAB for, and holds equity in, Interline Therapeutics, Umbra Therapeutics, Rapafusyn Pharmaceuticals and Ananke Therapeutics. He also holds equity in Jnana Therapeutics and consults for Matchpoint Therapeutics. The Center for Protein Degradation at DFCI receives research funding from Deerfield. E. S. F. is a founder, scientific advisory board (SAB) member, and equity holder of Civetta Therapeutics, Lighthorse Therapeutics, Proximity Therapeutics, and Neomorph, Inc. (board of directors). E. S. F. is an equity holder and SAB member for Avilar Therapeutics and Photys Therapeutics and a consultant to Novartis, Sanofi, EcoR1 Capital, and Deerfield. The Fischer lab receives or has received research funding from Deerfield, Novartis, Ajax, Interline, Voronoi, and Astellas. KAD is a consultant for Kronos Bio and Neomorph Inc. J. A. M. is a founder, equity holder, and advisor to Entact Bio, serves on the SAB of 908 Devices, and receives or has received sponsored research funding from Vertex, AstraZeneca, Taiho, Springworks and TUO Therapeutics.

## Supplementary Material

CB-004-D3CB00103B-s001
