# Supplementary material for: Development of a covalent cereblon-based PROTAC employing a fluorosulfate warhead
Source: RSC Chem Biol. 2023 Aug 31;4(11):906–12. doi: 10.1039/d3cb00103b (PMC10619143; doi:10.1039/d3cb00103b)
Supplement: CB-004-D3CB00103B-s001 [file CB-004-D3CB00103B-s001.pdf]

Electronic Supplementary Material (ESI) for RSC Chemical Biology.
This journal is © The Royal Society of Chemistry 2023

Electronic Supplementary Information

Development of a covalent CRBN PROTAC employing a fluorosulfate warhead

Radosław P. Nowak,[a,b,c]* Leah Ragosta,[a] Fidel Huerta,[a] Hu Liu,[a,b] Scott B. Ficarro,[d,e] Justin T. Cruite,[a,b] Rebecca J. Metivier,[c] Katherine A. Donovan,[b,c] Jarrod A. Marto,[d,e] Eric S. Fischer,[a,b,c] Breanna L. Zerfas[a,b] and Lyn H. Jones[a,b]*

[a.] *Center for Protein Degradation, Dana-Farber Cancer Institute, Boston MA, USA*
[b.] *Department of Biological Chemistry and Molecular Pharmacology, Harvard Medical School, Boston MA, USA*
[c.] *Department of Cancer Biology, Dana-Farber Cancer Institute, Boston MA, USA*
[d.] *Department of Cancer Biology, Department of Oncologic Pathology, and Blais Proteomics Center, Dana-Farber Cancer Institute, Boston MA, USA*
[e.] *Department of Pathology, Brigham and Women's Hospital, Harvard Medical School, Boston, MA 02115, USA*
Corresponding authors: lyn_jones@dfci.harvard.edu, nowak@crystal.harvard.edu

Contents	Page
Figure S1	2
Figure S2	3
Figure S3	3
General synthetic methods	4
Hazards	5
Synthesis of FS-ARV-825 covalent CRBN PROTAC	5
Cellular BRD4 HiBiT assay	22
Cellular IKZF1 HiBiT assay	22
Cellular GSPT1-GFP/mCherry degradation assay using Operetta	23
Cellular CRBN NanoBRET engagement assay	25
Kinetic readout of cellular CRBN NanoBRET engagement assay	26
MOLT4 Cell Titer Glo (CTG) viability assay	27
Purification of CRBN-DDB1 complex	27
CRBN labeling and peptide mapping MS	28
Quantitative degradation proteomics	29
References	31

A

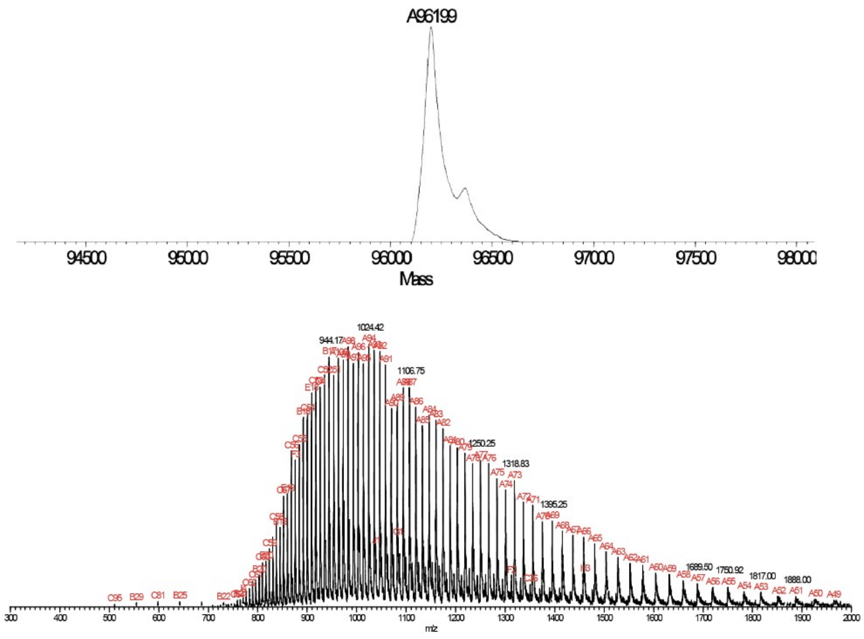

B

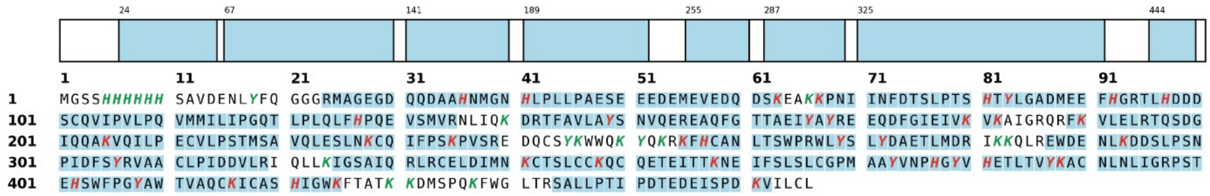

Figure S1. A. Intact mass spectrum of CRBN-DDB1ΔB treated with FS-ARV-825 (10 µM, 24h, RT). DDB1ΔB shows no labelling with FS-ARV-825. Deconvoluted spectra as well as m/z ratios as depicted. **B.** Coverage of the peptide mapping on cereblon after trypsin digest and LC-MS/MS as in A.

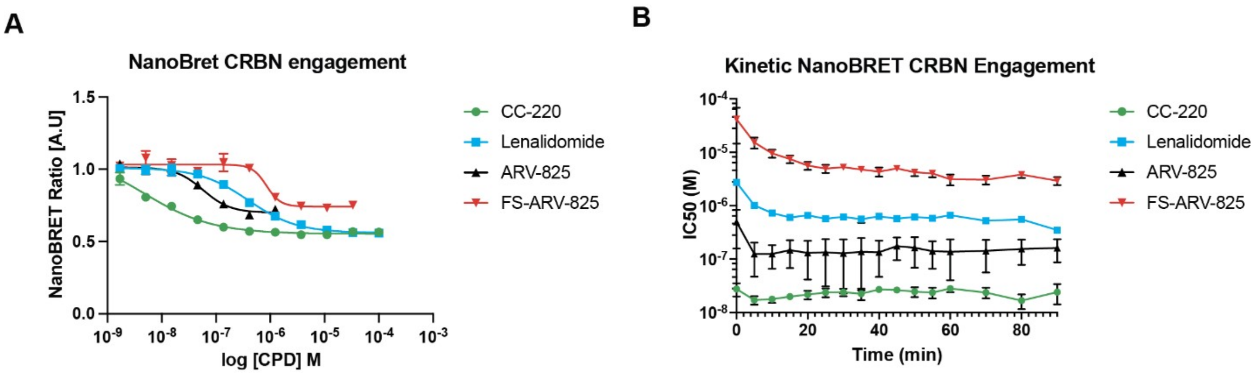

Figure S2. A. NanoBRET cellular cereblon engagement assay. NanoBRET signal at 520/450 nm was measured to quantify the displacement of BODIPY-lenalidomide probe in HEK293T cells stably expressing NanoLuc-CRBN following compound treatment. Data are shown as mean ± s.d. of two replicates (N=2). **B.** NanoBRET cellular cereblon engagement assay in a kinetic mode. Changes in the IC_{50} values were monitored over 90 minutes until the steady state. Data are shown as mean ± s.d. of two replicates (N=2).

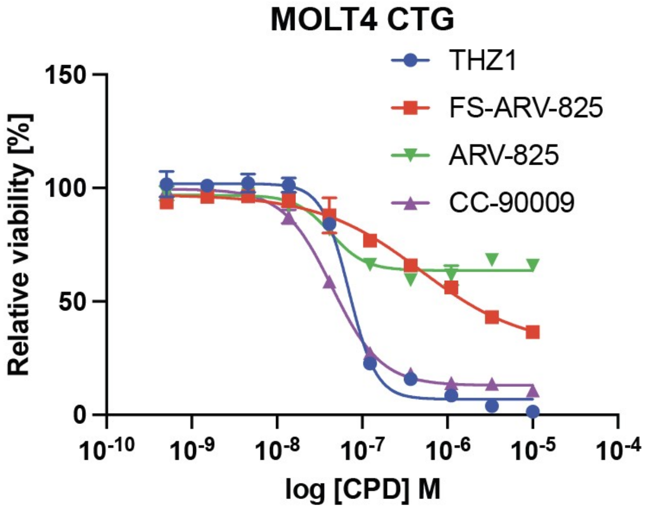

Figure S3. MOLT4 Cell Titer Glo (CTG) assay. MOLT4 cells were treated for 24h and the viability measured with CTG assay (Promega). Data is shown as mean ± s.d. of two replicates (N=2).

General synthetic methods

Unless otherwise indicated, reagents and solvents were used as received from commercial suppliers. All reactions were monitored using a Shimadzu® LC-20AD high performance liquid chromatography/mass spectrometry (HPLC/MS) system using Kinetex® BEH C18 column (2.1 x 30 mm, 5 μm particle size). Detection methods were diode array (DAD). MS mode was positive electrospray ionization. MS range was 100-1000. HPLC method A: the gradient was 5-95% B in 1.50 min. 5% B in 0.01 min, 5-95% B (0.01 - 0.70 min), 95% B (0.70 - 1.15 min), 5% B in 1.16 min with a hold at 5% B for 0.34 min; solvent A = 0.04% trifluoroacetic acid in H_2O; solvent B = 0.02% trifluoroacetic acid in acetonitrile; flow rate: 1.5 mL/min. Purification of reaction products was carried out by flash chromatography using CombiFlash®Rf with Biotage - Isolera® normal-phase silica flash columns; or Waters® high performance liquid chromatography (HPLC) system using Phenomenex Luna C18 (80*30mm*3μm): solvent gradient 0% to 99% acetonitrile in H_2O (0.1% trifluoroacetic acid (TFA) as additive); flow rate: 25 mL/min, or Phenomenex Luna C18 (100*30mm*5μm): solvent gradient 0% to 99% acetonitrile in H_2O (0.2% formic acid (FA) as additive); flow rate: 25 mL/min, or Phenomenex Luna C18 (80*30mm*3μm): solvent gradient 0% to 99% acetonitrile in H_2O (0.04% hydrochloric acid (HCl) as additive); flow rate: 25 mL/min. The purity of all compounds was over 95% and was analyzed with Shimadzu® HPLC system. ^1H NMR and ^{13}C NMR spectra were obtained using Bruker Avance III spectrometers (400 MHz for ^1H, and 100 MHz for ^{13}C). Chemical shifts are reported relative to deuterated methanol (δ = 3.31) or dimethyl sulfoxide (DMSO) (δ = 2.50) for ^1H NMR. Spectra are given in ppm (δ) and as br = broad, s = singlet, d = doublet, t = triplet, q = quartet, m = multiplet and coupling constants (J) are reported in Hertz.

Hazards

All cereblon modulators should be treated as teratogens.

Synthesis of FS-ARV825 covalent CRBN PROTAC

Synthesis of (9S)-7-(4-chlorophenyl)-9-[2-[4-[2-[2-[2-[2-[[2-(2,6-dioxo-3-piperidyl)-6-fluorosulfonyloxy-1,3-dioxo-isoindolin-4-yl]amino]ethoxy]ethoxy]ethoxy]ethoxy]anilino]-2-oxo-ethyl]-4,5,13-trimethyl-3-thia-1,8,11,12-tetrazatricyclo[8.3.0.02,6]trideca-2(6),4,7,10,12-pentaene

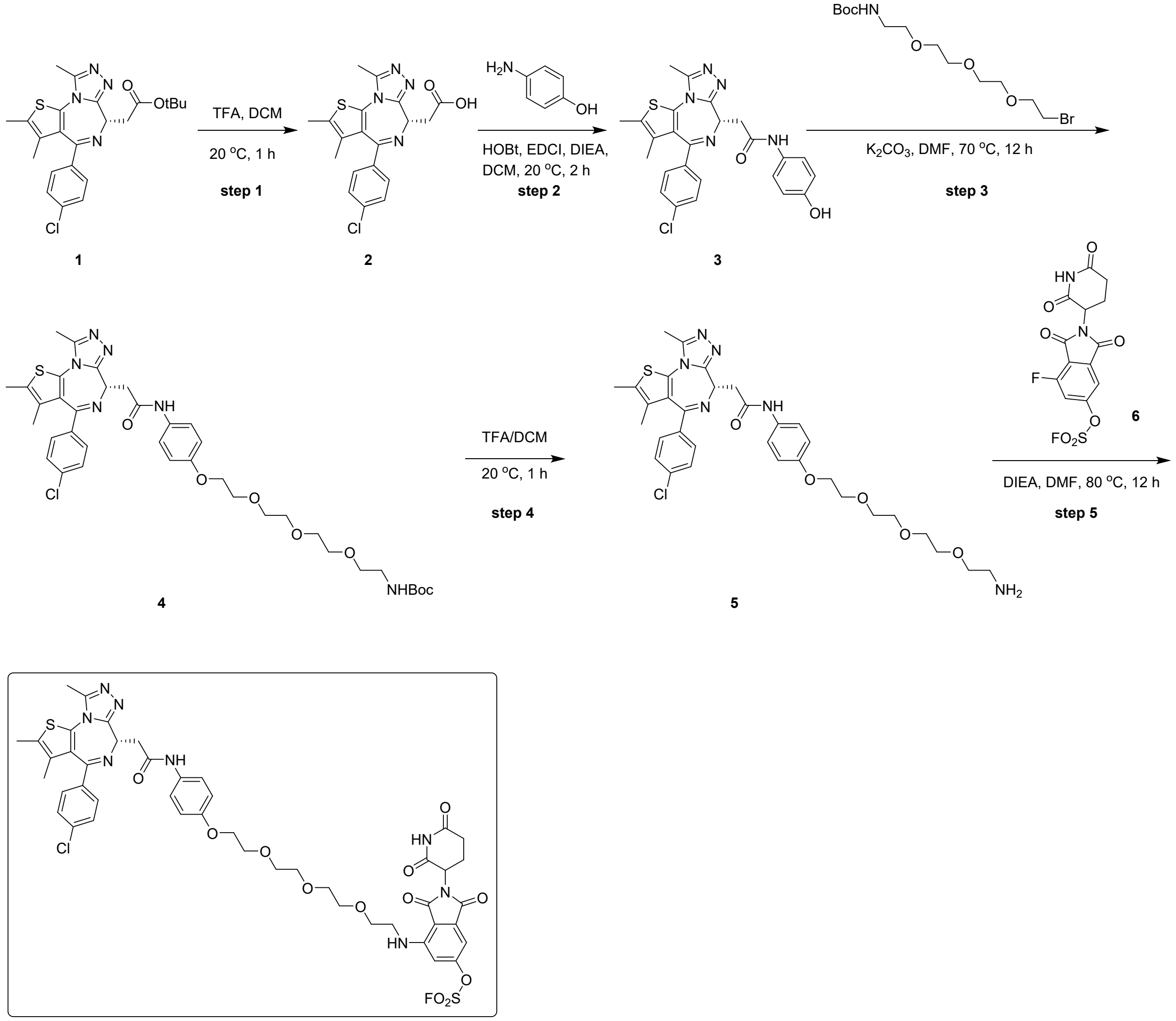

FS-ARV825

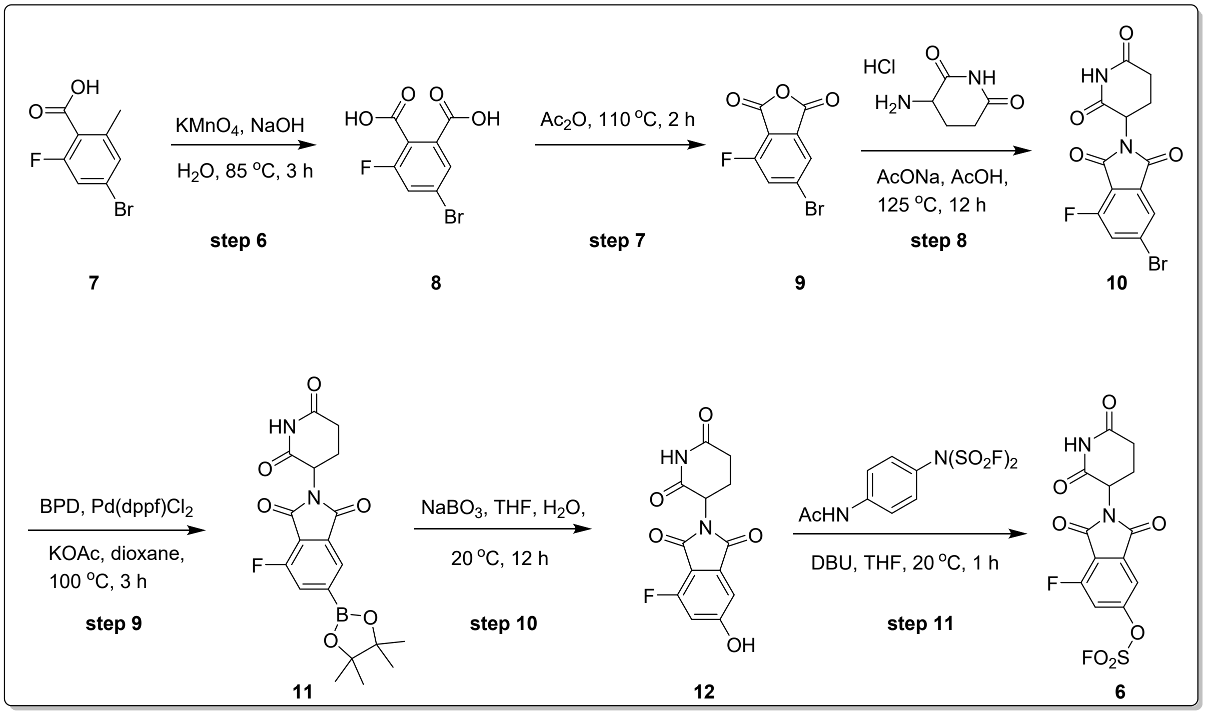

Step 1:

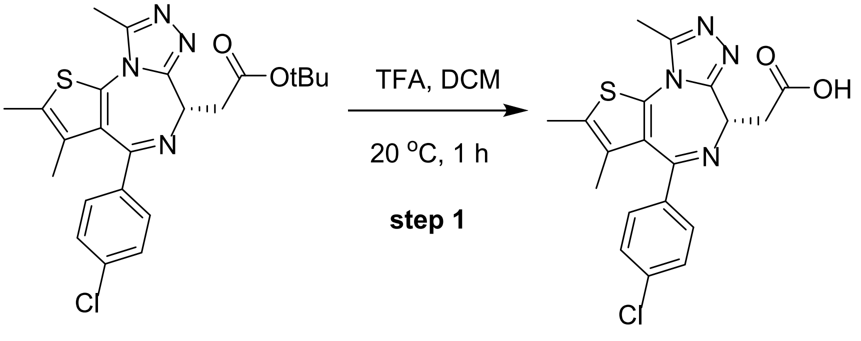

2-[(9S)-7-(4-chlorophenyl)-4,5,13-trimethyl-3-thia-1,8,11,12-tetrazatricyclo[8.3.0.02,6]trideca-2(6),4,7,10,12-pentaen-9-yl]acetic acid (2)

To a solution of tert-butyl2-[(9S)-7-(4-chlorophenyl)-4,5,13-trimethyl-3-thia-1,8,11,12-tetrazatricyclo[8.3.0.02,6]trideca-2(6),4,7,10,12-pentaen-9-yl]acetate (500 mg, 1.09 mmol, 1 eq) in DCM (5 mL) was added TFA (7.70 g, 67.53 mmol, 5.0 mL, 61.7 eq). The mixture was stirred at 20 °C for 1 hr. The reaction mixture was concentrated under reduced pressure to afford title

compound **2** (0.5 g, crude) as yellow oil, which was used into the next step without further purification.

Step 2:

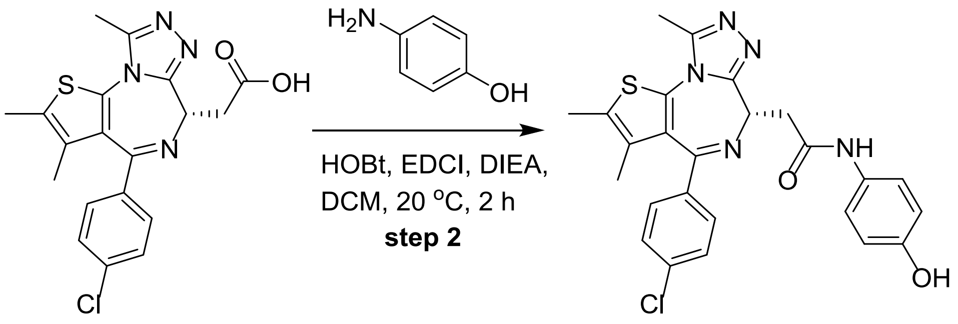

2-[(9S)-7-(4-chlorophenyl)-4,5,13-trimethyl-3-thia-1,8,11,12-tetrazatricyclo[8.3.0.02,6]trideca-2(6),4,7,10,12-pentaen-9-yl]-N-(4-hydroxyphenyl)acetamide (3)

To a solution of 2-[(9S)-7-(4-chlorophenyl)-4,5,13-trimethyl-3-thia-1,8,11,12-tetrazatricyclo[8.3.0.02,6]trideca-2(6),4,7,10,12-pentaen-9-yl]acetic acid (480 mg, 1.20 mmol, 1 eq) and 4-aminophenol (457 mg, 4.19 mmol, 653 uL, 3.5 eq) in DCM (24 mL) was added DIEA (1.24 g, 9.58 mmol, 1.67 mL, 8 eq), HOBt (243 mg, 1.80 mmol, 1.5 eq) and EDCI (344 mg, 1.80 mmol, 1.5 eq). The mixture was stirred at 20 °C for 2 h. The reaction mixture was concentrated under reduced pressure to give a residue. The residue was purified by column chromatography (SiO_2, Ethyl acetate/Ethyl alcohol=1/0 to 10/1) to afford title compound **3** (0.55 g, 1.12 mmol, 93% yield) as a yellow solid.

^{1}H NMR: (400 MHz, DMSO-d_6)

δ = 10.09 (s, 1H), 9.21 (s, 1H), 7.53 (s, 2H), 7.51-7.43 (m, 4H), 6.76 (d, *J* = 8.8 Hz, 2H), 4.69-4.59 (m, 1H), 3.50 (br d, *J* = 6.9 Hz, 2H), 2.66 (s, 3H), 2.48 (s, 3H), 1.69 (s, 3H)

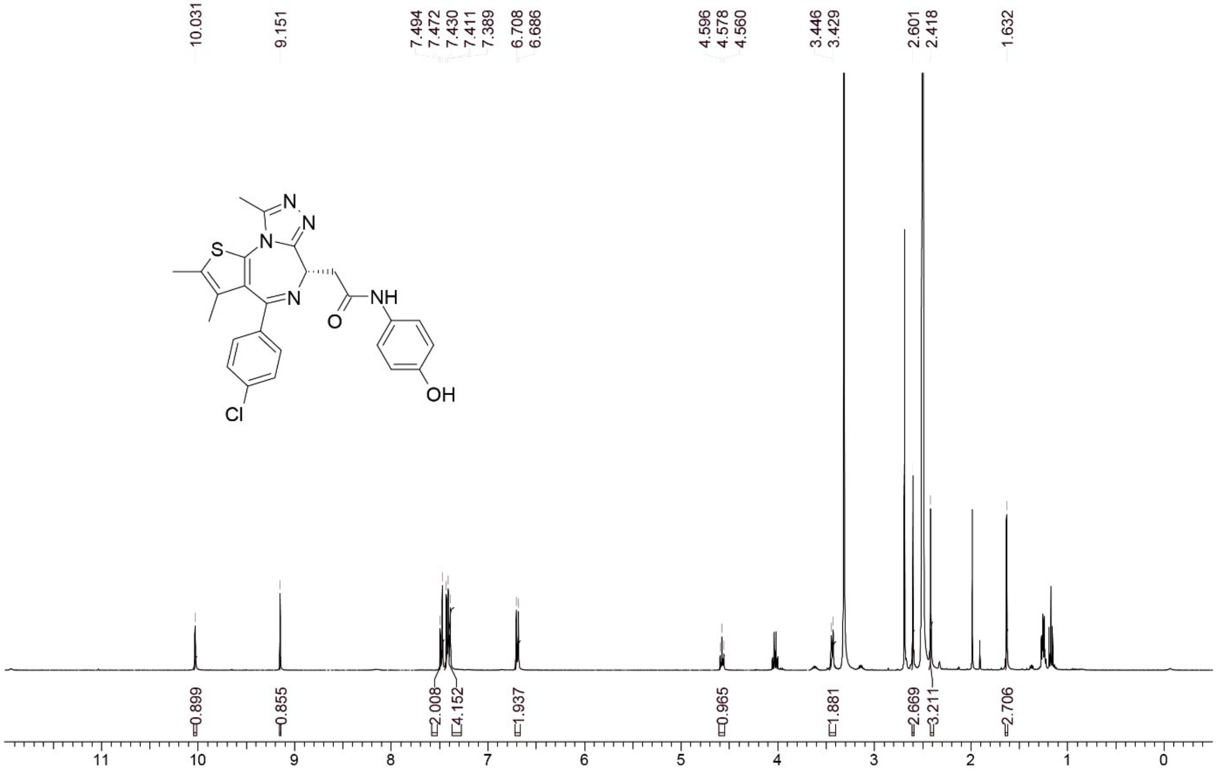

^{1}H NMR trace for compound 3.

Step 3:

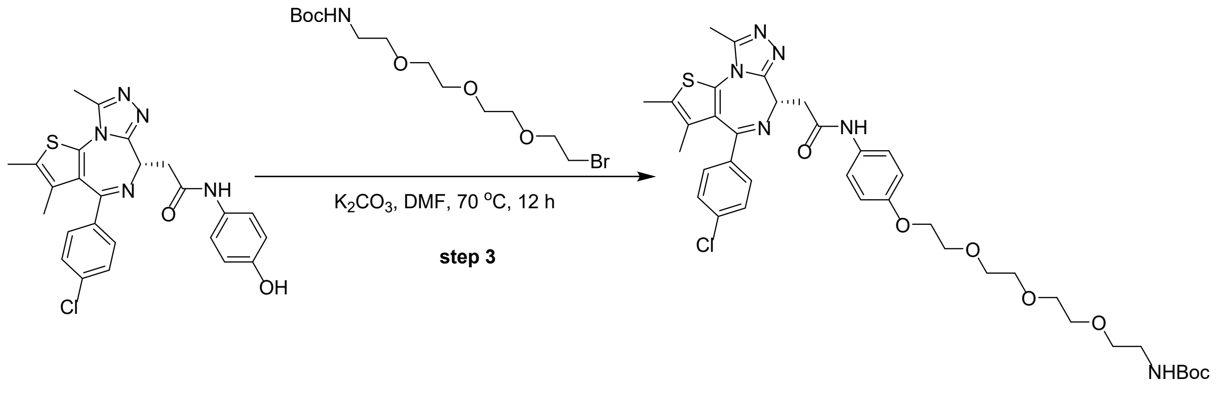

tert-butyl N-[2-[2-[2-[2-[4-[[2-[(9S)-7-(4-chlorophenyl)-4,5,13-trimethyl-3-thia-1,8,11,12-tetrazatricyclo[8.3.0.02,6]trideca-2(6),4,7,10,12-pentaen-9-yl]acetyl]amino]phenoxy]ethoxy]ethoxy]ethoxy]ethyl]carbamate (4)

To a solution of 2-[(9S)-7-(4-chlorophenyl)-4,5,13-trimethyl-3-thia-1,8,11,12-tetrazatricyclo[8.3.0.02,6]trideca-2(6),4,7,10,12-pentaen-9-yl]-N-(4-hydroxyphenyl)acetamide (300 mg, 610 µmol, 1 eq) and tert-butyl N-[2-[2-[2-(2-bromoethoxy)ethoxy]ethoxy]ethyl]carbamate (217 mg, 610 µmol, 1 eq) in DMF (7.5 mL) was added K_2CO_3 (253 mg, 1.83 mmol, 3 eq). The mixture was stirred at 70 °C for 12 h. The reaction mixture was poured into H_2O (20 mL), and extracted with EtOAc (20 mLx3). The combined organic layers were washed with brine (20 mL x 2), dried over Na_2SO_4, filtered and concentrated under reduced pressure to give a residue. The residue was purified by column chromatography (SiO_2, MeOH/DCM=1/50 to 1/30) to afford title compound **4** (0.2g, 43% yield) as a brown solid.

^{1}H NMR: (400 MHz, DMSO-d_6)

δ = 10.17 (s, 1H), 7.62 - 7.41 (m, 6H), 6.75 (br d, *J* = 5.4 Hz, 2H), 4.63-4.55 (m, 1H), 3.78 - 3.69 (m, 2H), 3.61-3.52 (m, 8H), 3.42-3.35 (m, 4H), 3.07 (m, 4H), 2.61 (s, 3H), 2.43 (s, 3H), 1.64 (s, 3H), 1.38 (s, 9H)

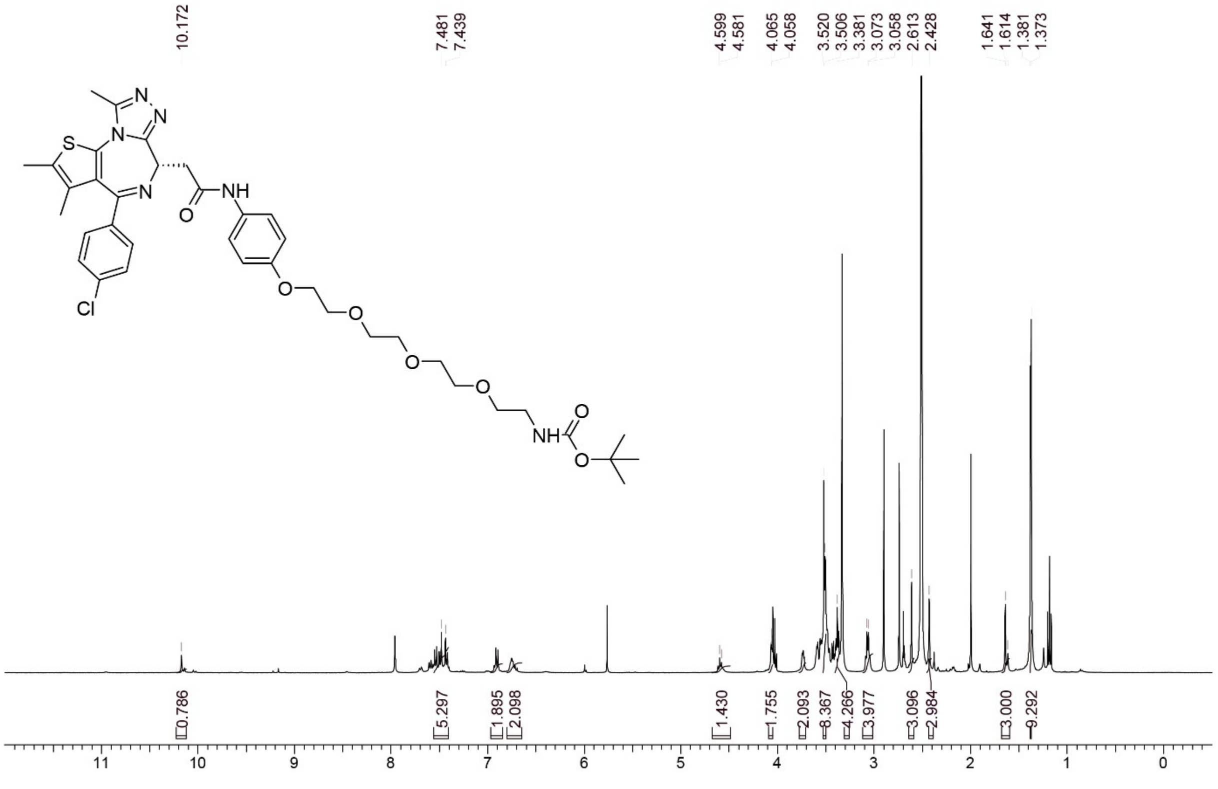

^{1}H NMR trace for compound 4.

Step 4:

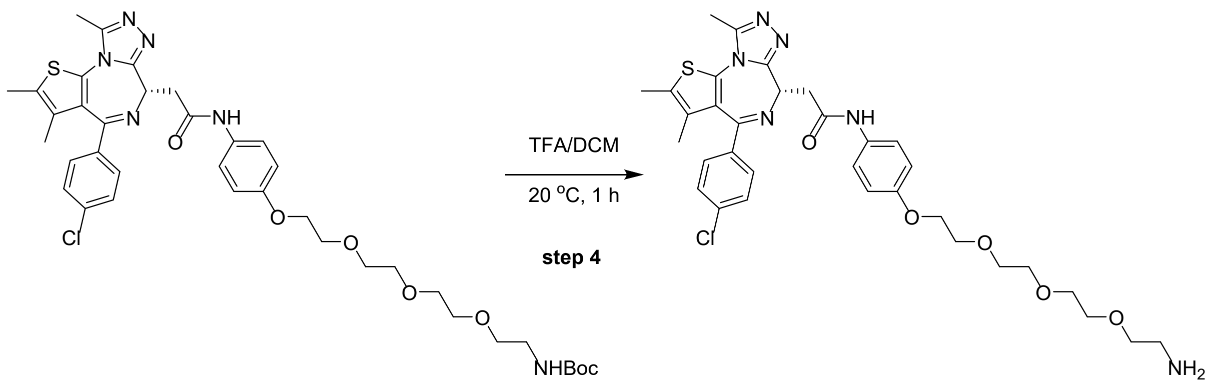

N-[4-[2-[2-[2-(2-aminoethoxy)ethoxy]ethoxy]ethoxy]phenyl]-2-[(9S)-7-(4-chlorophenyl)-4,5,13-trimethyl-3-thia-1,8,11,12-tetrazatricyclo[8.3.0.02,6]trideca-2(6),4,7,10,12-pentaen-9-yl]acetamide (5)

To a solution of tert-butyl N-[2-[2-[2-[2-[4-[[2-[(9S)-7-(4-chlorophenyl)-4,5,13-trimethyl-3-thia-1,8,11,12-tetrazatricyclo[8.3.0.02,6]trideca-2(6),4,7,10,12-pentaen-9-yl]acetyl]amino]phenoxy]ethoxy]ethoxy]ethoxy]ethyl]carbamate (0.2 g, 261 µmol, 1 eq) in DCM (2 mL) was added TFA (308 mg, 2.70 mmol, 0.2 mL, 10.4 eq). The mixture was stirred at 20 °C for 1 h. The reaction mixture was concentrated under reduced pressure to afford title compound **5** (0.22 g, crude, TFA salt) as brown oil, which was used into the next step without further purification.

Step 6:

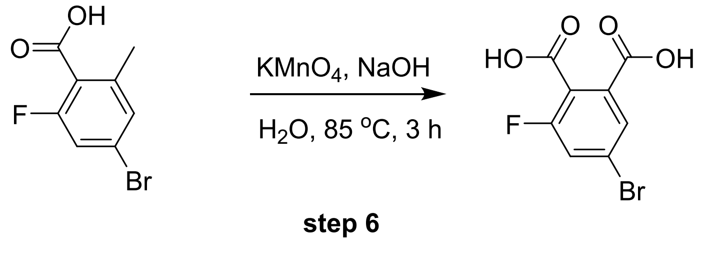

5-bromo-3-fluoro-phthalic acid (8)

To a solution of 4-bromo-2-fluoro-6-methyl-benzoic acid (12 g, 51.49 mmol, 1 eq) in NaOH (1 M, 154.5 mL, 3 eq) was added $KMnO_4$ (65.10 g, 411.96 mmol, 8 eq) portion-wise during 3 h at 85 °C. The mixture was stirred at 85 °C for 3 h. The reaction mixture was filtered and the filter cake was washed with H_2O (100 mL). The filtrate was adjusted pH to 1 with 12 N HCl, and extracted with EtOAc (100 mL x 3). The combined organic layers were washed with brine (100 mL x 2) and dried over Na_2SO_4, filtered and concentrated under reduced pressure to afford title compound **8** (8 g, 30.42 mmol, 59% yield) as a white solid, which was used into the next step without further purification. The structure was confirmed by ^{1}H NMR.

^{1}H NMR: (400 MHz, DMSO-d_6)

δ = 15.19-12.13 (m, 1H), 7.98 (dd, *J* = 1.9, 8.9 Hz, 1H), 7.90-7.83 (m, 1H)

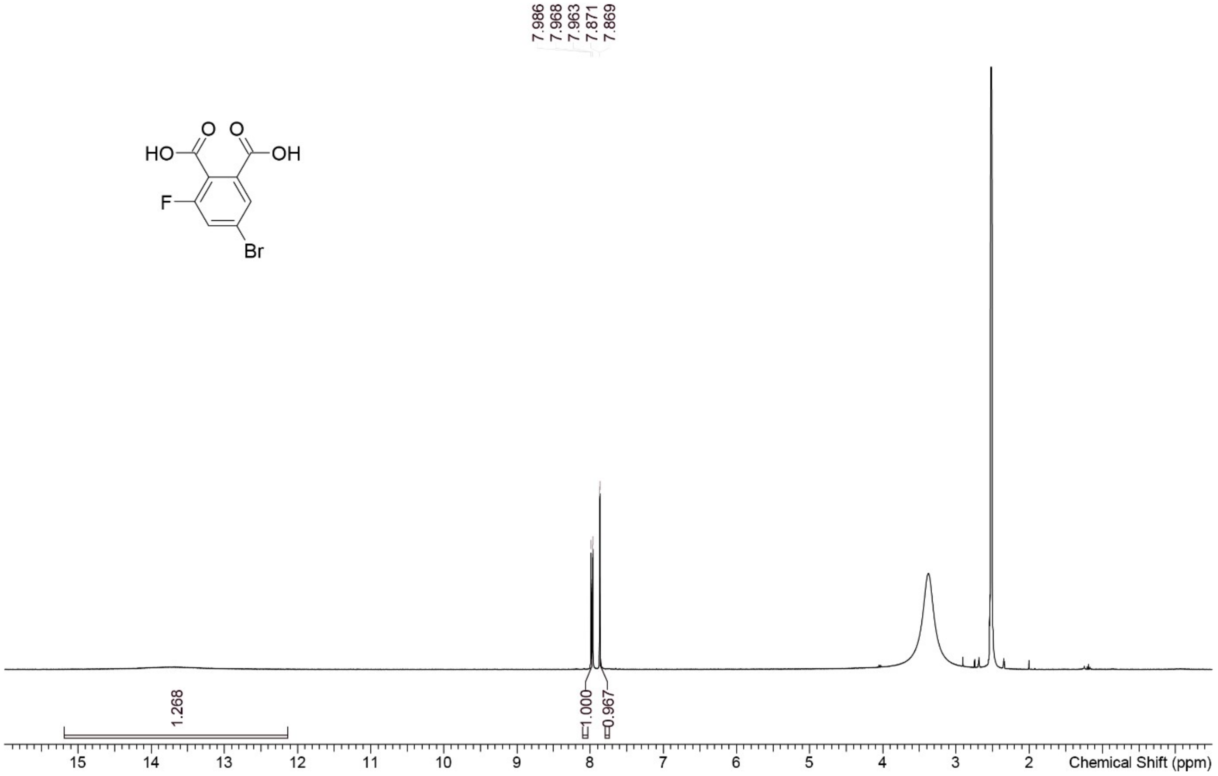

^{1}H NMR trace for compound 8.

Step 7:

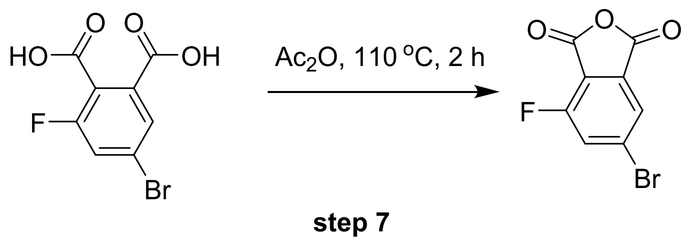

6-bromo-4-fluoro-isobenzofuran-1,3-dione (9)

A solution of 5-bromo-3-fluoro-phthalic acid (8 g, 30.42 mmol, 1 eq) in Ac_2O (80 mL) was stirred at 110 °C for 2 h. The reaction mixture was concentrated under reduced pressure to afford title compound **9** (7.3 g, crude) as yellow oil, which was used into the next step without further purification.

Step 8:

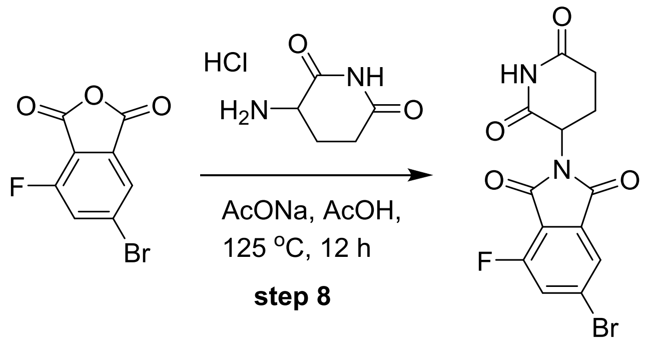

6-bromo-2-(2,6-dioxo-3-piperidyl)-4-fluoro-isoindoline-1,3-dione (10)

To a solution of 6-bromo-4-fluoro-isobenzofuran-1,3-dione (7.3 g, 29.80 mmol, 1 eq) in AcOH (100 mL) was added NaOAc (4.89 g, 59.59 mmol, 2 eq) and 3-aminopiperidine-2,6-dione (4.90 g, 29.80 mmol, 1 eq, HCl). The mixture was stirred at 125 °C for 12 h. The reaction mixture was poured into H_2O 100 mL, while yellow solid formed. Then the solid was filtered and washed by H_2O (20 mL x 2). The cake was collected and dried under reduced pressure to afford title

compound **10** (8 g, crude) as a yellow solid, which was used into the next step without further purification.

^{1}H NMR: (400 MHz, DMSO-d_6)

δ = 11.15 (s, 1H), 8.13 (dd, *J* = 1.1, 8.8 Hz, 1H), 8.01 (d, *J* = 1.1 Hz, 1H), 5.14 (dd, *J* = 5.5, 12.9 Hz, 1H), 2.93 - 2.80 (m, 1H), 2.62 - 2.54 (m, 1H), 2.45 (m, 1H), 2.10 - 1.96 (m, 1H)

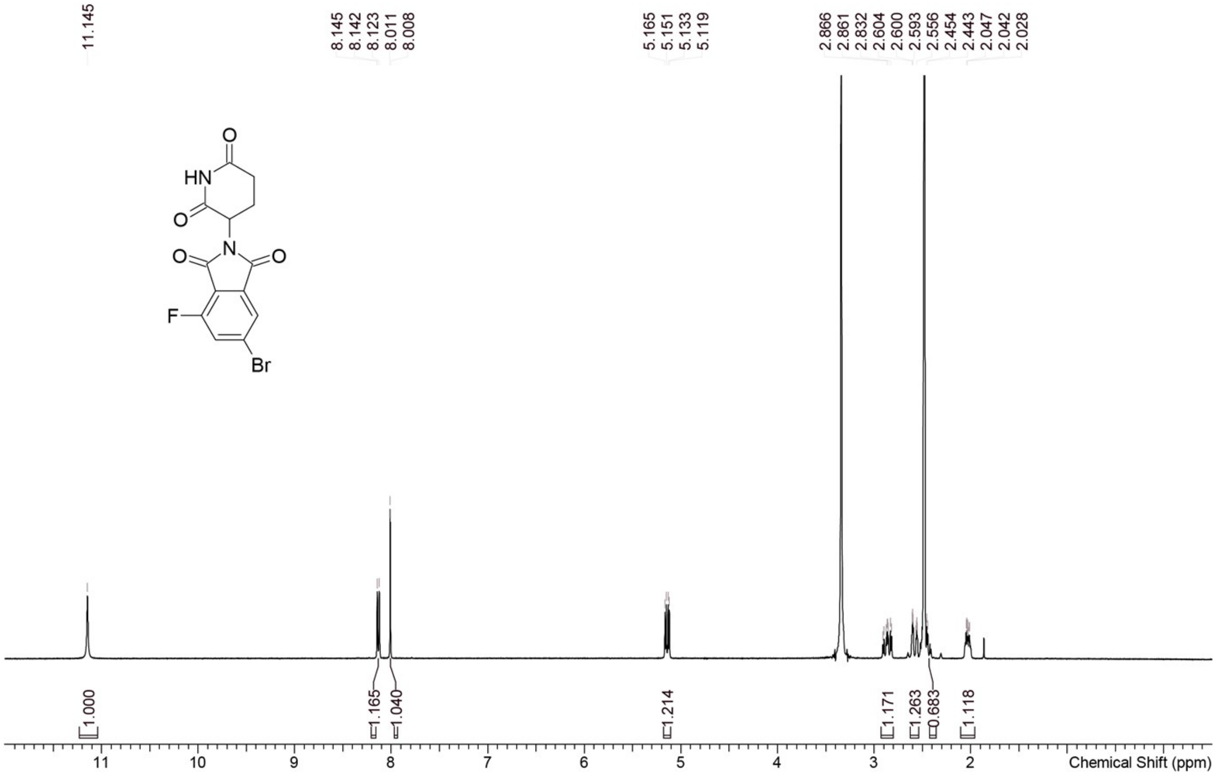

^{1}H NMR trace for compound 10.

Step 9:

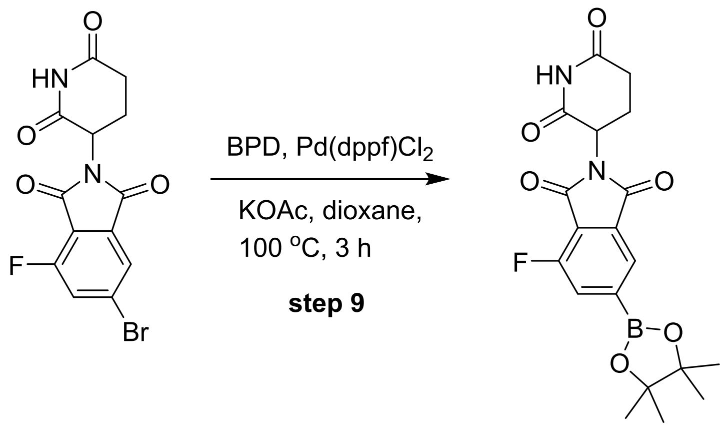

2-(2,6-dioxo-3-piperidyl)-4-fluoro-6-(4,4,5,5-tetramethyl-1,3,2-dioxaborolan-2-yl)isoindoline-1,3-dione (11)

A mixture of 6-bromo-2-(2,6-dioxo-3-piperidyl)-4-fluoro-isoindoline-1,3-dione (4 g, 11.26 mmol, 1 eq), BPD (8.58 g, 33.79 mmol, 3 eq), Pd(dppf)Cl_2 (824 mg, 1.13 mmol, 0.1 eq), KOAc (2.21 g, 22.53 mmol, 2 eq) in dioxane (50 mL) was degassed and purged with N_2 for 3 times, and then the mixture was stirred at 100 °C for 3 h under N_2 atmosphere. The reaction mixture was then poured into H_2O 50 mL and extracted with EtOAc (50 mL x 2). The combined organic layers were washed with brine (50 mL x 2), dried over Na_2SO_4, filtered and concentrated under reduced pressure to give a residue. The crude product was triturated with PE/EA (4/1, 50 mL), and filtered. The cake was dried under reduced pressure to afford title compound (2.3 g, 48% yield, 95% purity) as a yellow solid.

^{1}H NMR: (400 MHz, DMSO-d_6)

δ = 11.16 (s, 1H), 8.03 - 7.62 (m, 2H), 5.18 (dd, *J* = 5.4, 12.9 Hz, 1H), 3.01 - 2.79 (m, 1H), 2.64 (m, 2H), 2.12 - 2.02 (m, 1H), 1.35 (s, 12H)

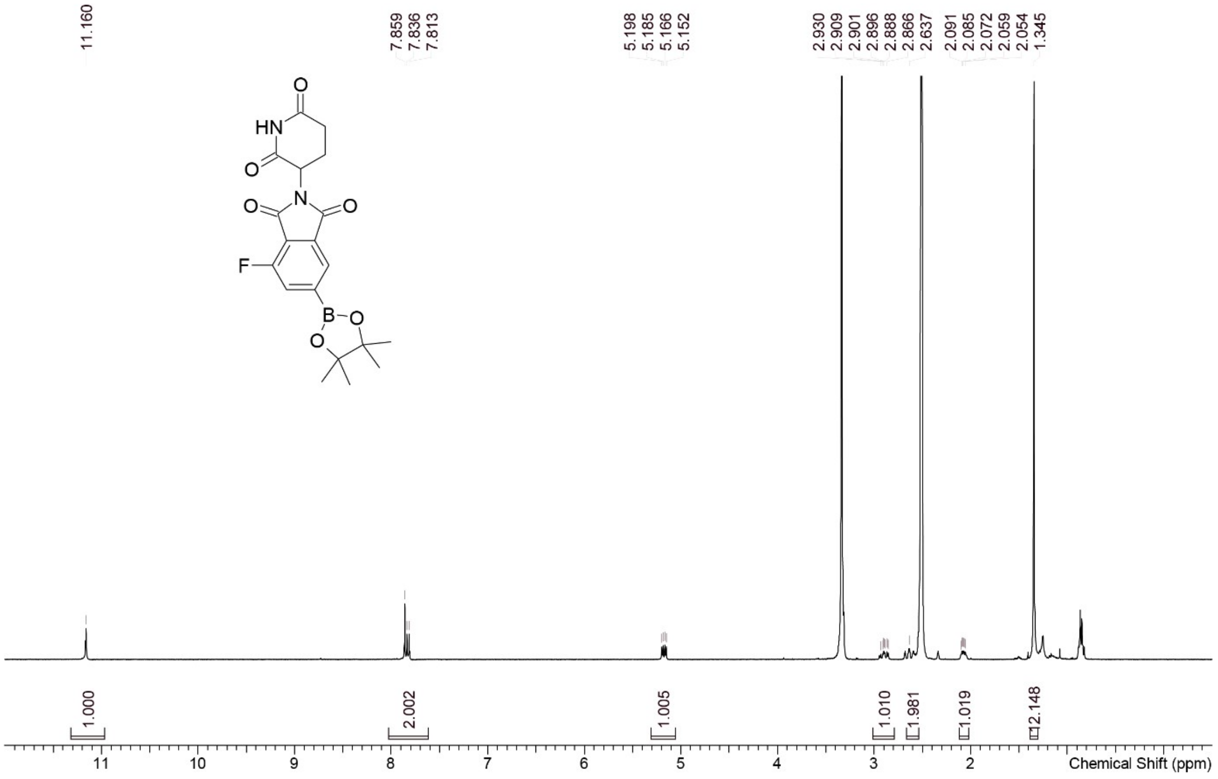

^{1}H NMR trace for compound 11.

Step 10:

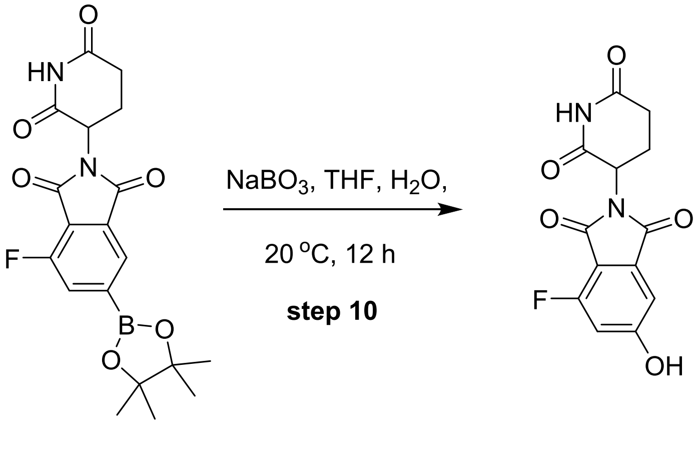

2-(2,6-dioxo-3-piperidyl)-4-fluoro-6-hydroxy-isoindoline-1,3-dione (12)

To a solution of 2-(2,6-dioxo-3-piperidyl)-4-fluoro-6-(4,4,5,5-tetramethyl-1,3,2-dioxaborolan-2-yl)isoindoline-1,3-dione (1 g, 2.49 mmol, 1 eq) in THF (20 mL) and H_2O (10 mL) was added sodium;3-oxidodioxaborirane;tetrahydrate (1.30 g, 8.45 mmol, 1.6 mL, 3.4 eq). The mixture was

stirred at 20 °C for 12 h. The reaction mixture was poured into H_2O (50 mL) and extracted with EtOAc (50 mL x 2). The combined organic layers were washed with brine (50 mL x 2), dried over Na_2SO_4, filtered and concentrated under reduced pressure to give a residue. The crude product was triturated with PE/EtOAc (4/1, 30 mL), and filtered. The cake was dried under reduced pressure to afford title compound (0.2 g, 25% yield, 90% purity) as a yellow solid. The purity was about 90% on ^1H NMR.

^{1}H NMR: (400 MHz, DMSO-d_6)

δ = 12.13 - 11.29 (m, 1H), 11.13 (s, 1H), 7.07 (d, *J* = 1.8 Hz, 1H), 6.95 (dd, *J* = 1.4, 11.0 Hz, 1H), 5.10 (dd, *J* = 5.4, 12.9 Hz, 1H), 2.97 - 2.82 (m, 1H), 2.66 - 2.49 (m, 2H), 2.09 - 2.01 (m, 1H)

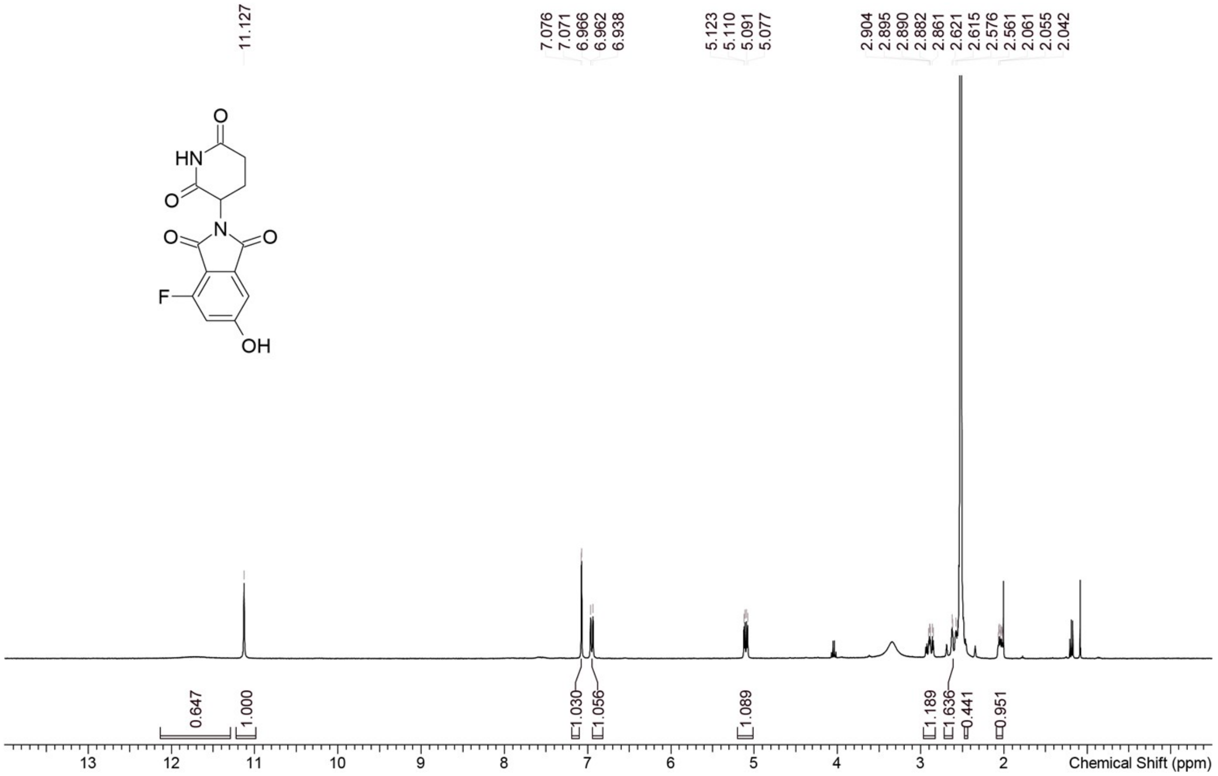

^{1}H NMR trace for compound 12.

Step 11:

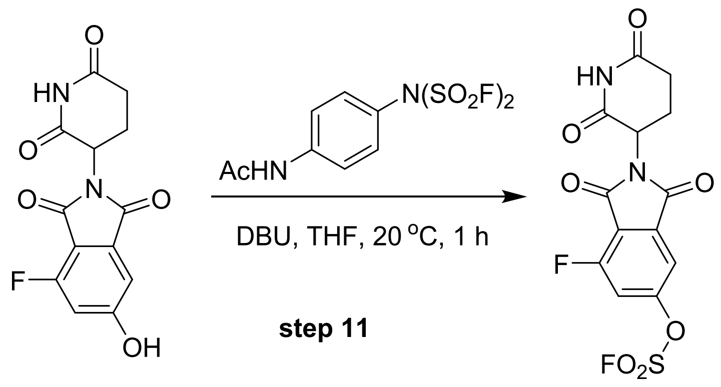

2-(2,6-dioxo-3-piperidyl)-4-fluoro-6-fluorosulfonyloxy-1,3-dioxo-isoindoline (6)

To a solution of 2-(2,6-dioxo-3-piperidyl)-4-fluoro-6-hydroxy-isoindoline-1,3-dione (0.15 g, 513 µmol, 1 eq) in THF (4 mL) was added DBU (55 mg, 359 µmol, 54 uL, 0.7 eq) and N-(4-acetamidophenyl)-N-fluorosulfonyl-sulfamoyl fluoride (161 mg, 513 µmol, 1 eq). The mixture was stirred at 20 °C for 1 h. The reaction mixture was concentrated under reduced pressure to afford title compound (0.18 g, crude) as brown oil, which was used into the next step without further purification.

Step 5:

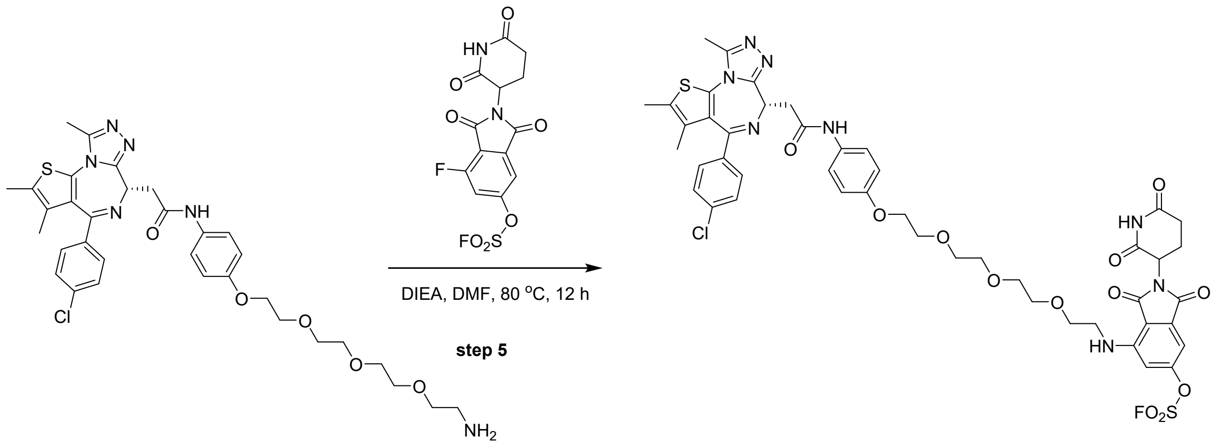

FS-ARV825

(9S)-7-(4-chlorophenyl)-9-[2-[4-[2-[2-[2-[2-[[2-(2,6-dioxo-3-piperidyl)-6-fluorosulfonyloxy-1,3-dioxo-isoindolin-4-yl]amino]ethoxy]ethoxy]ethoxy]ethoxy]anilino]-2-oxo-ethyl]-4,5,13-

trimethyl-3-thia-1,8,11,12-tetrazatricyclo[8.3.0.02,6]trideca-2(6),4,7,10,12-pentaene (FS-ARV825)

To a solution of N-[4-[2-[2-[2-(2-aminoethoxy)ethoxy]ethoxy]ethoxy]phenyl]-2-[(9S)-7-(4-chlorophenyl)-4,5,13-trimethyl-3-thia-1,8,11,12-tetrazatricyclo[8.3.0.02,6]trideca-2(6),4,7,10,12-pentaen-9-yl]acetamide (0.22 g, 281 µmol, 1 eq, TFA salt) in DMF (2 mL) was added 2-(2,6-dioxo-3-piperidyl)-4-fluoro-6-fluorosulfonyloxy-1,3-dioxo-isoindoline (0.18 g, 481 µmol, 1.7 eq) and DIEA (109 mg, 845 µmol, 147 uL, 3 eq). The mixture was stirred at 80 °C for 12 h. The reaction mixture was concentrated under reduced pressure to give a residue. The residue was purified by prep-HPLC (column: Phenomenex Luna 80*30mm*3um;mobile phase: [water(HCl)-ACN];B%: 40%-70%,8min) to afford title compound (7 mg, 2% yield, 91% purity, HCl salt) as a yellow solid.

The purity was 91% on HPLC. The structure was confirmed by ^{1}H NMR and ^{19}F NMR and MS.

MS: ($M+H^{+}$): 1021.4

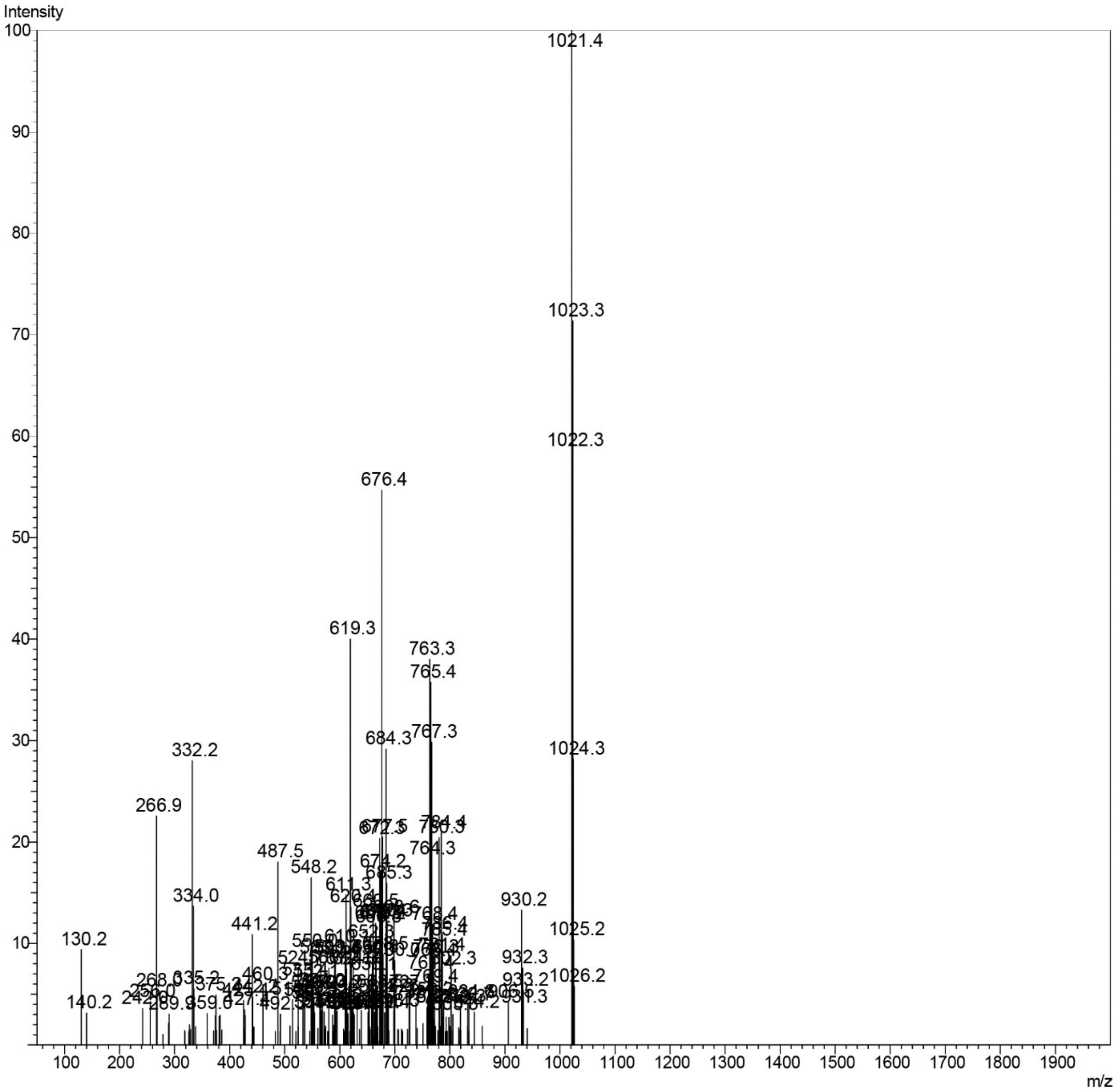

HPLC: 91.066@3.810 min (10-80% ACN in H_2O, 4.5 min)

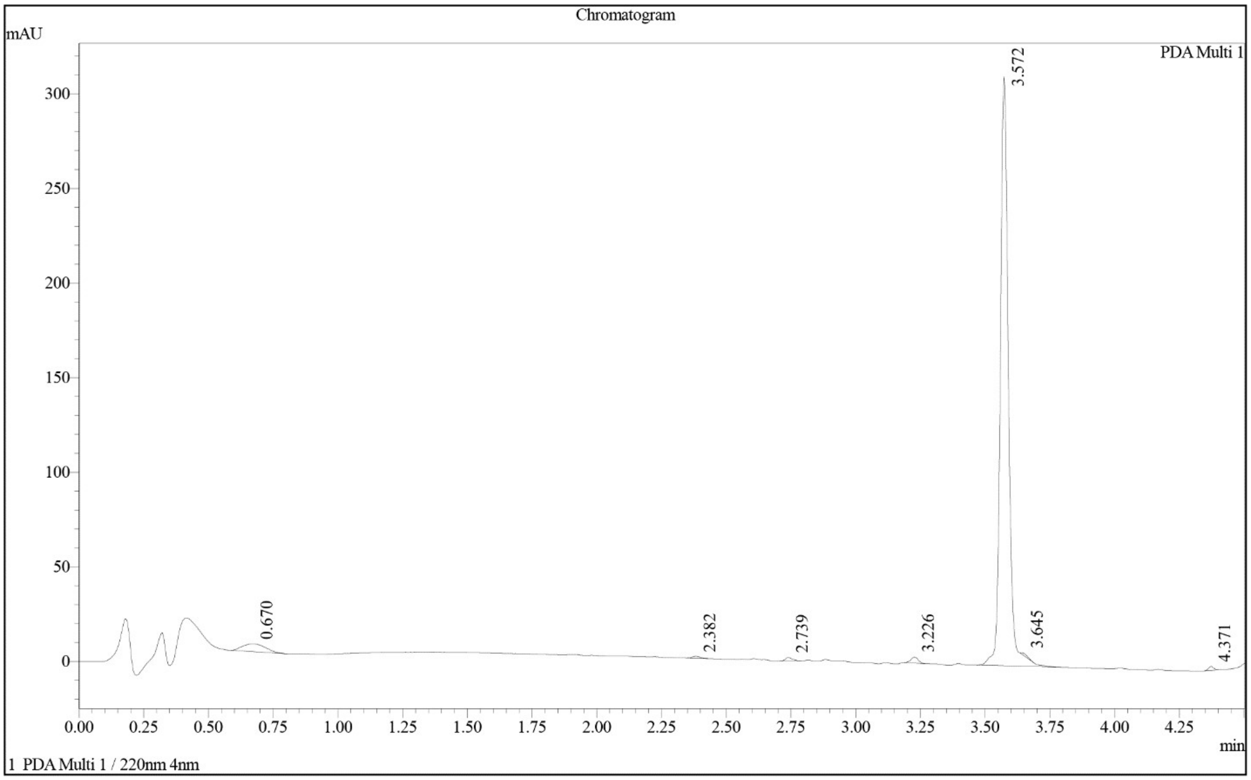

Integration Result

PDA Ch1 220nm 4nm

Peak#	Ret. Time	Height	Height %	USP Width	Area	Area %
1	0.670	4102	1.264	0.167	25873	3.606
2	2.382	993	0.306	0.059	2122	0.296
3	2.739	1781	0.549	0.054	3434	0.479
4	3.226	3229	0.995	0.056	6202	0.864
5	3.572	311370	95.939	0.058	675388	94.119
6	3.645	1027	0.316	0.047	1641	0.229
7	4.371	2048	0.631	0.043	2932	0.409

^{1}H NMR: (400MHz, DMSO-d_6)

δ = 11.10 (s, 1H), 10.15 (s, 1H), 7.51 - 7.43 (m, 4H), 7.41 - 7.34 (m, 3H), 7.17 (d, *J* = 1.5 Hz, 1H), 6.94 (br d, *J* = 3.3 Hz, 1H), 6.84 (d, *J* = 9.0 Hz, 2H), 5.05 (dd, *J* = 5.4, 12.8 Hz, 1H), 4.58 (t, *J* = 7.0 Hz, 1H), 4.03 - 3.94 (m, 2H), 3.68 - 3.63 (m, 2H), 3.58 - 3.55 (m, 2H), 3.48 (br d, *J* = 5.0 Hz, 12H), 2.87 - 2.79 (m, 1H), 2.60 - 2.48 (m, 5H), 2.38 (s, 3H), 2.04 - 1.96 (m, 1H), 1.59 (s, 3H).

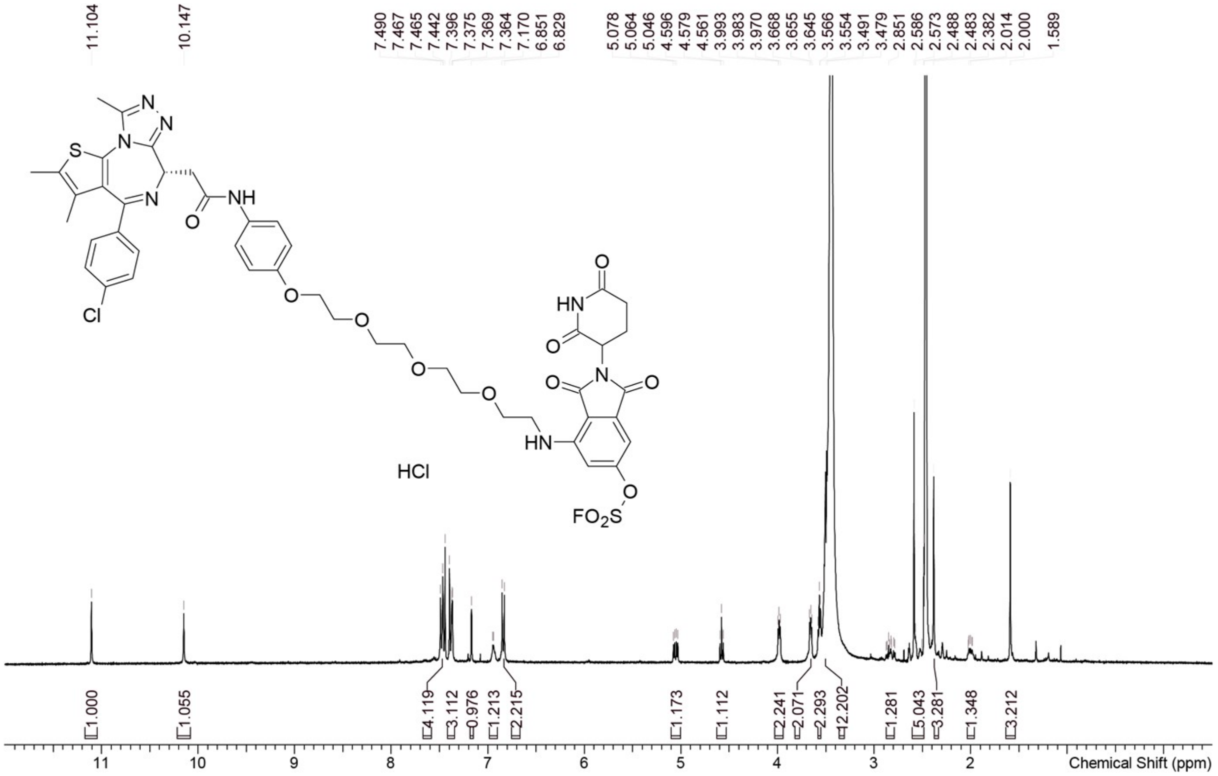

^{19}F NMR: (400MHz, DMSO-d$_6$)

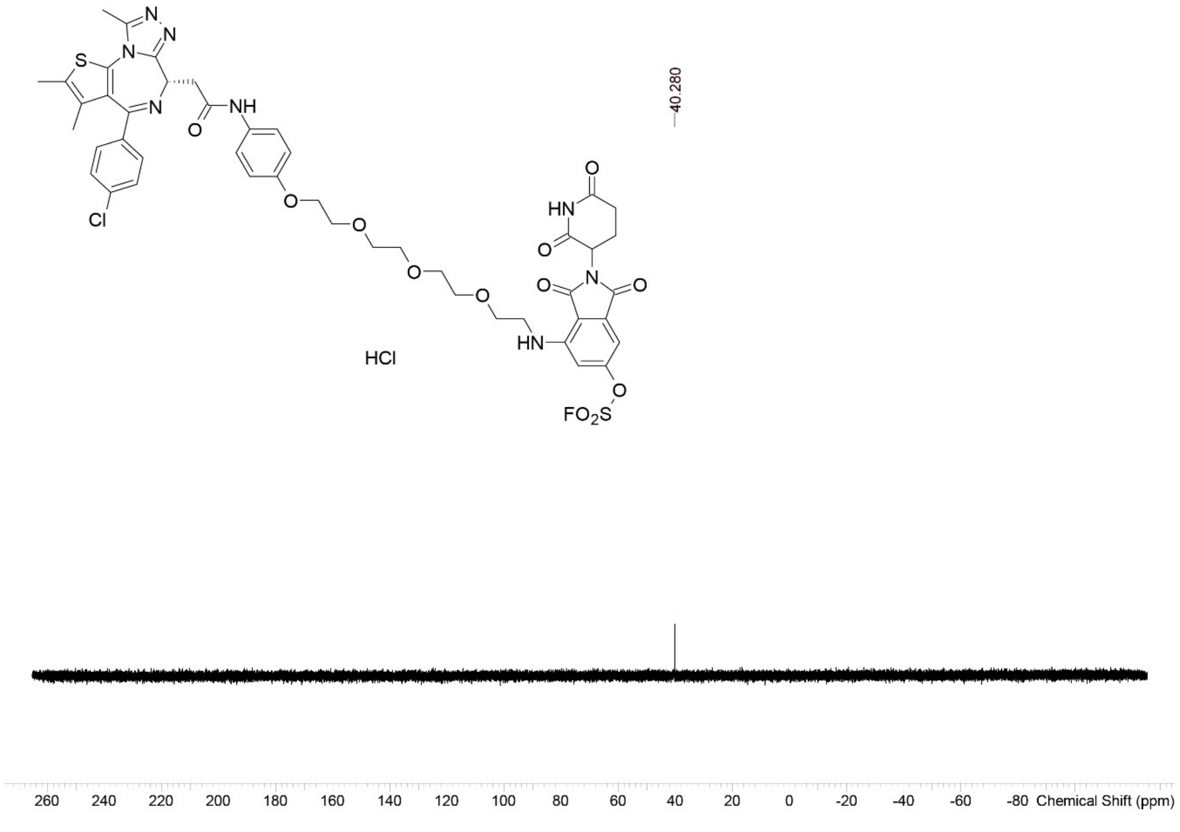

Cellular BRD4 HiBiT assay

HEK293T cells with N-terminal HiBiT-SpyTag knock in at the BRD4 locus were prepared as previously described.[1] HiBiT-SpyTag BRD4 HEK293T cells were seeded at 5,000 cells per well in a 384-well plate (Thermo Scientific, 164610) at 50 µL per well in DMEM media (Thermo Fisher Scientific, 11965118) containing 10% FBS and incubated overnight. Compounds were dispensed using D300e Digital Dispenser (HP) and normalized to 0.5% DMSO. Cells were pretreated with MLN4924 for 2 hours before treatment or directly with the PROTAC degraders ARV-825 (HY-16954, MedChemExpress) and FS-ARV-825, or co-treated with CC-92480 for 5 or 24 hours. HiBiT assay was performed as described in the manufacturer protocol (Promega, N3030). 12.5 µL of premixed detection reagent was added to each well of the 384 well assay plate using ClipTip Pipettes (Thermo Fisher) and incubated for 15 min. The luminescence signal was quantified using PHERAstar® FSX microplate reader (BMG Labtech). Data was analyzed using GraphPad Prism software with curve fitting performed using variable slope equation.

Cells in washout experiment with HiBiT-SpyTag-BRD4 cells were first treated for 5h with FS-ARV-825, ARV-825 or dBET6, followed by 3 cycles of PBS wash, where media was removed with a plate washer and PBS gently added with ClipTip multichannel pipette (Thermo Fischer), and 24h recovery incubation in DMEM media containing 10 % FBS supplemented with 1 µM of CC-92480 (used to block CRBN activity). The control plate was run as described above, but no washout was applied making the total incubation time 29h (5h +24h). The assay was performed and analyzed as described above.

Cellular IKZF1 HiBiT assay

To run the assay, compounds were first dispensed ranging from 10 µM in 3-fold dilution into 384-well white flat bottom TC-treated plates (Corning, 3570). Then 4000 cells per well in 50

µL of media were plated. Cells were mixed, centrifuged at 500 rpm for 1 min, and incubated for 24h at 37ºC, 5% CO_2. Before reading, plates were equilibrated to room temperature, while HiBiT lytic reagent was prepared according to manufacturer's instructions (Promega): for 10 mL of reagent, dilute 200 µL HiBiT substrate and 100 µL of LgBiT protein in 10 mL of lytic buffer. 12.5 µL of prepared reagent was added to each well (1:4 dilution). Plates were shaken at room temperature for 10 min under aluminum foil cover, then read on an EnVision plate reader (Perkin Elmer). For data analysis, DMSO-treated samples were averaged, and %DMSO treated was calculated for each test sample. Data was analyzed using GraphPad Prism 9 and dose response curves were fitted using Variable Slope equation model.

<u>Cellular GSPT1-GFP/mCherry degradation assay using Operetta</u>

GSPT1 GFP/mCherry (Flp293T based) cells were maintained in DMEM high glucose media (11965118, Thermo Fisher) supplemented with 10% FBS (FSP500, ExCellBio), 1% penicillin/streptomycin (SV30010, Hyclone) and 5 µg/mL hygromycin B (10687010, Invitrogen) in T-75 tissue culture flasks. To prepare an assay plate media was aspirated carefully from T-75 flask with confluent cells, following by a wash with 10 mL dPBS. After aspiration of dPBS 1 mL of 0.05% Trypsin (25300062, Invitrogen) was added to the cells on the plate and swirled around to evenly disperse on the surface and incubated in the hood for 2 min at room temperature. 10 mL of fresh culture media was then added to the plate with the trypsin and striped cells by pipetting up and down around the plate. Afterwards all the cells were transferred to a 50 mL Falcon tube, spun down for 5 mins at 1000 rpm. After spinning down, media was aspirated, and the cell pellet resuspend in 5 mL assay media (FluoroBrite DMEM (A1896701, Invitrogen) supplemented with 10% FBS and 1% penicillin/streptomycin). At this point cells were counted and made up to 40 mL of 0.16 x 10^6/mL cells in assay media (8000 cells/well). Cell mix was plated in the 384 well plate

(781090, Corning) by adding 50 µL/well using multi-drop dispenser (Multi-dropCombi, Thermo Fisher). After plating, the assay plate was spun down in centrifuge for 5 mins at 500xg and incubated at 37ºC, 5% CO_2 overnight, allowing the cells to adhere. Compounds were dispensed the next day using Labcyte acoustic dispenser (POD810), 100 µM top, 3 times dilution, and 11-point dose response in duplicate with the final DMSO concentration of 1%. Positive control (CC-885) had a top concentration of 0.1 µM. After dispensing compounds, the assay plate was returned to incubator for 5h. After 5h incubation, 15 µl of 16% formaldehyde (28908, Pierce) was dispense into each well directly using multi-drop (Multi-dropCombi, Thermo Fisher) using standard tube dispensing cassette (24072670, Thermo Fisher) with the final concentration of formaldehyde of 4%. The assay plate was then spun down at 500 rpm, for 1 min, then incubated in the dark for 15 mins. Following the incubation media was decanted by inverting plate and flicking out media over a container. Finally, 50 µl of dPBS (18007771, Corning) was added into each well and the plate was read plate using Operetta CLS (HH1600, Perkin Elmer). The imaging was performed with non-confocal optical mode using 10x Air, NA 0.3 objective with binning set to 2. The channels were defined as: Ch1 - Digital Phase Contrast, 10 ms acquisition, 10% intensity and 0 µm offset; Ch2 – EGFP, 200 ms acquisition, 100% intensity and 0 µm offset; Ch3 – mCherry, 200 ms acquisition, 100% intensity and 0 µm offset. The EGFP intensity threshold was set to 10,000. For data analysis the %GFP positive cells were calculated as:

%GFP positive cells = (# Cells with more than 10,000 EGFP) / (# Cells with mCherry) * 100%.

Data was reported as %DMSO treatment, where:

%DMSO treatment = (%GFP positive cells sample) / (Average %GFP positive cells MAX) * 100%

and analyzed using GraphPad Prism 9 and dose response curves were fitted using Variable Slope equation.

Cellular CRBN NanoBRET Engagement Assay

The assay was performed as previously described[2]. HEK293T stably expressing NanoLuc-CRBN were cultured in DMEM (Gibco, Life Technologies) supplemented with 10% FBS and 1 μg/mL puromycin to maintain stable NanoLuc-CRBN expression. To run the assay, 21 mL of cells resuspended at 2 x 10^5 viable cells/mL in Opti-MEM I (Gibco, Life Technologies) were mixed with 600 μL of the CRBN engagement tracer (stock at 10 μM in 31.25% PEG-400, 12.5 mM HEPES, pH 7.5, filtered using a 0.22 μm nitrocellulose membrane) to reach final concentration of the tracer at 278 nM. The cell-tracer mixture was then plated in a white/opaque cell culture treated 384-well plate (Corning, 3570) at 50 μL/well. After plating, the assay plate was centrifuged (500 x g, 5 min) and covered in aluminum foil. Compounds for testing were added to the plate using a D300e Digital Dispenser (HP) in duplicate 12-pt titrations from a 10 mM stock in DMSO, with DMSO normalized to 1% total volume. The plate was then placed in an incubator at 37 °C, 5% CO_2 for two hours. After incubation, the plate was removed and set on the bench to cool to room temperature (~10-15 min). The NanoLuc substrate (500X solution) and extracellular inHiBiTor (1500X solution) were diluted in Opti-MEM I (Gibco, Life Technologies) to prepare a 3X solution. This was then added to each well (25 μL/well). The plate was read on a Pherastar FSX microplate reader with simultaneous dual emission capabilities to read 384-well plates at 450 and 520 nm. The NanoBRET ratio was calculated by dividing the signal at 520 nm by the signal at 450 nm for each sample. Duplicate points were averaged and plotted against [compound, M] to generate an EC_{50} curve and analyzed using GraphPad Prism 9 and dose response curves were fitted using Variable Slope equation. The Nluc substrate and extracellular inHiBiTor were purchased as a kit

from Promega Corporation and used as is from the box - Promega NanoBRET Nano-Glo Substrate/InHiBiTor; Promega Catalog number N2161 for 10,000 assay kit.

Cellular CRBN NanoBRET Kinetic Engagement Assay

The assay was performed using the same cell line as reported for the end-point NanoBRET assay.[3] To run the assay, 12 mL of cells resuspended at 4 x 10^5 viable cells/mL in Opti-MEM I without phenol red (Gibco, Life Technologies) + 10% FBS were mixed with 300 µL of the CRBN engagement tracer (stock at 10 µM in 31.25% PEG-400, 12.5 mM HEPES, pH 7.5, filtered using a 0.22 µm nitrocellulose membrane) to reach a final concentration of the tracer at 244 nM. The cell-tracer mixture was then plated in a white/opaque cell culture treated 384-well plate (Thermo Scientific – Nunc, 164610) at 25 µL/well. After plating, the assay plate was centrifuged (500xg, 5 min) and covered in aluminum foil. The plate was placed in an incubator at 37 °C, 5% CO2 for three hours. Immediately before use, a 2X solution of Intracellular TE Nano-Glo Vivazine/inHiBiTor (Promega, N2200) was prepared by adding 240 µL 100X stock vivazine and 16 µL of extracellular inHiBiTor (30 mM stock in DMSO) to a final volume of 12 mL Opti-MEM I without phenol red (Gibco, Life Technologies) + 10% FBS. This solution was then added to the assay plate at 25 µL/well and the plate was centrifuged (500 x g, 5 min). Compounds for testing were added to the plate using a D300e Digital Dispenser (HP) in duplicate 12-pt titrations from a 10 mM stock in DMSO, with DMSO normalized to 1% total volume. The plate was read on a Pherastar FSX microplate reader with simultaneous dual emission capabilities to read 384-well plates at 450 and 520 nm while incubating at 37 °C. The NanoBRET ratio was calculated by dividing the signal at 520 nm by the signal at 450 nm for each sample. Duplicate points were averaged and plotted against [compound, M] and analyzed using GraphPad Prism 9.

MOLT4 Cell Titer Glo (CTG) viability assay

Compounds dissolved in DMSO were dispensed using LabCyte Acoustic Dispenser (POD810) in a 10-point dose response, in duplicates, with 0.5% final DMSO concentration, into 384-well TC treated assay plates (Greiner, 781080) and the assay ready plates were centrifuged at 500 rpm for 1 min. THZ-1 was used as a positive control on each assay plate. MOLT-4 cells were seeded in the assay ready plates at 10,000 cells per well at 50 μL volume per well in RPMI 1640 media (Invitrogen, 22400105) supplemented with 10% FBS (ExCellBio, FSP500) using multi-drop dispenser (Thermo Scientific, Milti-drop Combi). Plates were incubated for 24h in mammalian incubator at 37°C, 5% CO_2. CellTiter-Glo® Luminescent Cell Viability Assay (CTG) assay was performed the following day. CTG reagents (Promega, G7573) were equilibrated to room temperature (RT) for 30 min and the assay plate was removed from the incubator and allowed to cool down to RT for 10 min. To run the assay, 12.5 μL of CTG reagent was added to each well using multi-drop dispenser (Thermo Scientific, Milti-drop Combi), the plate was centrifuges at 500 rpm for 1 min, covered in aluminum foil, shaken on a plate shaker for 10 min, centrifuged for 1 min at 500 rpm and read on EnVision plate reader (Perkin Elmer, 2104-0010). The data was analyzed using GraphPad Prism 9 and dose response curves were fitted using Variable Slope equation.

Purification of CRBN-DDB1ΔB complex

Human CRBN and DDB1ΔB were cloned into pAC-derived vectors[4] and recombinant protein complex was co-expressed as StrepTag-Avi-CRBN and His_6-Spy-DDB1ΔB fusions in *Trichoplusia ni* High-Five insect cells using the baculovirus expression system (Invitrogen). Cells were lysed by sonication in 50 mM Tris-HCl pH 8.0, 200 mM NaCl, 1 mM TCEP, 1 mM PMSF and 1x protease inHiBiTor cocktail (Sigma). Following ultracentrifugation and filtration, the soluble fraction was incubated with Strep-Tactin XT Superflow high-capacity resin for 1 hr at 4°C

and eluted with buffer (50 mM Tris-HCl pH 8.0, 200 mM NaCl, 1 mM TCEP) containing 100 mM biotin. The complex was further purified via anion exchange chromatography (Poros 50HQ) and size exclusion chromatography in 25 mM HEPES pH 7.4, 200 mM NaCl and 1 mM TCEP. Fractions containing the purified CRBN-DDB1 complex were concentrated using ultrafiltration (Millipore), flash frozen in liquid nitrogen, and stored at −80°C.

Cereblon Labeling and Peptide Mapping MS

Biotinylated $BRD4_{BD1}$ or $BRD4_{BD2}$ were purified as described before[5]. The hsCRBN-DDB1 complex was treated with DMSO or an equimolar concentration of compounds or the $BRD4_{BD1}$ or $BRD4_{BD2}$ all at 10 μM concentration for 24 hrs at room temperature before being analyzed by LC−MS using an HPLC system (Shimadzu, Marlborough, MA) interfaced to an LTQ ion trap mass spectrometer (ThermoFisher Scientific, San Jose, CA). After injecting 5 μg, proteins were desalted for four minutes on column with 100% A and then eluted with an HPLC gradient (0−100% B in 1 min; A = 0.2 M acetic acid in water; B = 0.2 M acetic acid in acetonitrile). The mass spectrometer was programmed to acquire full scan mass spectra (m/z 300−2000) in profile mode (spray voltage = 4.5 kV). Mass spectra were deconvoluted using MagTran software version 1.03b2.

To identify the site of covalent modification, compound treated hsCRBN-DDB1 complex was reduced with 10 mM dithiothreitol for 30 min at 56 °C, alkylated with 25 mM iodoacetamide for 30 min at room temperature, and digested with trypsin (Promega, Madison, WI) overnight at 37 °C. Peptides were desalted using C18 (SOLA, ThermoFisher Scientific, Madison, WI), and analyzed by nanoflow LC-MS/MS using a NanoAcquity UPLC system interfaced to a QExactive HF mass spectrometer (ThermoFisher Scientific).[6] Peptides were resolved on a self-packed analytical column (50 cm x 30 μm packed with 5 μm Monitor C18) using a gradient (2-50% B in

60 minutes, A=0.1% formic acid, B=0.1% formic acid in acetonitrile) and introduced to the mass spectrometer by electrospray (spray voltage = 5 kV). The mass spectrometer was operated in data dependent mode and the ten most abundant ions in each MS scan (m/z 300− 2000, 120K resolution, 1E6 target, 50 ms max fill time) were subjected to MS/MS (15K resolution, 1E5 target, 100 ms max fill time). Dynamic exclusion was enabled with a repeat count of 1 and an exclusion duration of 30s. Raw mass spectrometry data files were converted to .mgf using Multiplierz software and searched against a custom database containing the hsCRBN-DDB1 sequences using Mascot version 2.6.2. Search parameters specified fixed carbamidomethylation of cysteine, variable methionine oxidation, and variable FS-ARV-825 modification of histidine, lysine, and tyrosine. Modified spectra were examined and figures prepared using mzStudio software.[7]

Quantitative degradation proteomics

MOLT-4 cells were treated with 1 μM of FS-ARV-825 in biological singlicate and DMSO vehicle control in biological triplicate for 5h. Cell lysis and Tandem Mass Tagged (TMT) tryptic peptides were prepared for LC-MS analysis following procedures published in Donovan et al 2018[8].

Data were collected using an Orbitrap Fusion Lumos mass spectrometer (Thermo Fisher Scientific, San Jose, CA, USA) coupled with a Proxeon EASY-nLC 1200 LC pump (Thermo Fisher Scientific). Peptides were separated on a 50 cm 75 μm inner diameter EasySpray ES903 microcapillary column (Thermo Fisher Scientific) using a 190 min gradient of 6 - 27% acetonitrile in 1.0% formic acid with a flow rate of 300 nL/min.

Each analysis used a MS3-based TMT method as described previously[9]. The data were acquired using a mass range of m/z 340 – 1350, resolution 120,000, AGC target 1×10^6, maximum injection time 100 ms, dynamic exclusion of 120 s for the peptide measurements in the Orbitrap. Data

dependent MS2 spectra were acquired in the ion trap with a normalized collision energy (NCE) set at 35%, AGC target set to 1.8×10^4 and a maximum injection time of 120 ms. MS3 scans were acquired in the Orbitrap with HCD collision energy set to 55%, AGC target set to 1.5×10^5, maximum injection time of 150 ms, resolution at 50,000 and with a maximum synchronous precursor selection (SPS) precursors set to 10.

Proteome Discoverer 2.5 (Thermo Fisher Scientific) was used for .RAW file processing and controlling peptide and protein level false discovery rates, assembling proteins from peptides, and protein quantification from peptides. MS/MS spectra were searched against a Swissprot human database (February 2020) with both the forward and reverse sequences as well as known contaminants such as human keratins. Database search criteria were as follows: tryptic with two missed cleavages, a precursor mass tolerance of 20 ppm, fragment ion mass tolerance of 0.6 Da, static alkylation of cysteine (57.02146 Da), static TMT labeling of lysine residues and N-termini of peptides (229.16293 Da), and variable oxidation of methionine (15.99491 Da). TMT reporter ion intensities were measured using a 0.003 Da window around the theoretical m/z for each reporter ion in the MS3 scan. The peptide spectral matches with poor quality MS3 spectra were excluded from quantitation (summed signal-to-noise across channels < 100 and precursor isolation specificity < 0.5), and the resulting data was filtered to only include proteins with a minimum of 2 unique peptides quantified. Reporter ion intensities were normalized and scaled using in-house scripts in the R framework[10]. Statistical analysis was carried out using the limma package within the R framework[11].

References

1. T. Tsang, F. Huerta, Y. Liu, J. Che, R. J. Metivier, S. Ferrao, K. A. Donovan, L. H. Jones, B. L. Zerfas and R. P. Nowak, *ACS Chem Biol*, 2023, **18**, 933-941.
2. J. T. Cruite, G. P. Dann, J. Che, K. A. Donovan, S. Ferrao, S. B. Ficarro, E. S. Fischer, N. S. Gray, F. Huerta, N. Kong, H. Liu, J. A. Marto, R. J. Metivier, R. P. Nowak, B. L. Zerfas and L. H. Jones, *RSC Chemical Biology*, 2022, DOI: 10.1039/D2CB00078D.
3. B. L. Zerfas, F. Huerta, H. Liu, G. Du, N. S. Gray, L. H. Jones and R. P. Nowak, in *Methods in Enzymology*, Academic Press, 2022, DOI: https://doi.org/10.1016/bs.mie.2022.08.013.
4. W. Abdulrahman, M. Uhring, I. Kolb-Cheynel, J. M. Garnier, D. Moras, N. Rochel, D. Busso and A. Poterszman, *Anal Biochem*, 2009, **385**, 383-385.
5. R. P. Nowak, S. L. DeAngelo, D. Buckley, Z. He, K. A. Donovan, J. An, N. Safaee, M. P. Jedrychowski, C. M. Ponthier, M. Ishoey, T. Zhang, J. D. Mancias, N. S. Gray, J. E. Bradner and E. S. Fischer, *Nat Chem Biol*, 2018, **14**, 706-714.
6. S. B. Ficarro, Y. Zhang, Y. Lu, A. R. Moghimi, M. Askenazi, E. Hyatt, E. D. Smith, L. Boyer, T. M. Schlaeger, C. J. Luckey and J. A. Marto, *Anal Chem*, 2009, **81**, 3440-3447.
7. S. B. Ficarro, W. M. Alexander and J. A. Marto, *Proteomes*, 2017, **5**.
8. K. A. Donovan, J. An, R. P. Nowak, J. C. Yuan, E. C. Fink, B. C. Berry, B. L. Ebert and E. S. Fischer, *Elife*, 2018, **7**.
9. G. C. McAlister, D. P. Nusinow, M. P. Jedrychowski, M. Wuhr, E. L. Huttlin, B. K. Erickson, R. Rad, W. Haas and S. P. Gygi, *Anal Chem*, 2014, **86**, 7150-7158.
10. R. C. Team, *Journal*, 2020.
11. M. E. Ritchie, B. Phipson, D. Wu, Y. Hu, C. W. Law, W. Shi and G. K. Smyth, *Nucleic Acids Res*, 2015, **43**, e47.
